# Risk factors for idiopathic pulmonary fibrosis: an umbrella review of meta-analyses and mendelian randomization analyses

**DOI:** 10.1186/s12890-026-04519-1

**Published:** 2026-07-22

**Authors:** Huanrong Ruan, Siyuan Lei, Jun Wang, Hulei Zhao, Xuezhong Zhou, Jiansheng Li

**Affiliations:** 1 Lung Disease Diagnosis and Treatment Center, National Medical Center, The First Affiliated Hospital of Henan University of Chinese Medicine, Zhengzhou, 450003 China; 2 Collaborative Innovation Center for Chinese Medicine and Respiratory Diseases Co-constructed by Henan Province & Education Ministry of P.R. China/Henan Key Laboratory of Chinese Medicine for Respiratory Diseases, Henan University of Chinese Medicine, Zhengzhou, 450046 China; 3 Institute of Medical Intelligence, School of Computer and Information Technology, Beijing Jiaotong University, Beijing, 100044 People’s Republic of China

**Keywords:** Idiopathic pulmonary fibrosis, Risk factors, Umbrella review, Meta-analyses, Mendelian randomization analyses

## Abstract

**Background:**

The incidence of idiopathic pulmonary fibrosis (IPF) is rising globally. Existing meta-analyses and Mendelian randomization (MR) studies have yielded inconsistent findings with uncertain evidence quality.

**Objectives:**

This study aimed to synthesize available evidence and assess its credibility for potential risk factors of IPF.

**Methods:**

We systematically searched 8 databases and selected meta-analyses and MR studies related to IPF risk factors from inception to March 11, 2025. We assessed the quality of meta-analyses using the AMSTAR-2 tool and evaluated MR studies using the STROBE-MR guidelines. We categorized the evidence into four levels and performed pooled analysis for risk factors reported in at least two MR studies.

**Results:**

A total of 8 meta-analyses were included, reporting 22 associations, mainly involving genetics, infections, occupational and environmental exposures, lifestyle, and clinical variables. According to the grading of evidence credibility, 12 were rated as suggestive associations, and 10 as weak associations. The overall credibility of the evidence is relatively low. The 36 MR studies investigated a total of 124 associations, with evidence grading showed 13 as robust, 90 as possible, 17 as suggestive, and 4 as insufficient. The pooled analysis identified 108 associations covering lifestyle, diet and nutrition, anthropometric indices, biomarkers, and clinical variables. The quality level of the included meta-analyses was low and critically low, while the MR studies strictly adhered to STROBE-MR, demonstrating good reporting quality.

**Conclusions:**

This umbrella review uncovers the evidence regarding the associations between IPF risk and factors such as genetics, viral infections, occupational and environmental exposures, lifestyle, and clinical variables through a comprehensive analysis of meta-analyses and MR studies. These findings provide an evidence base for IPF risk factor research and support clinical decisions and public health strategies.

**PROSPERO registration number:**

CRD42024575341.

**Supplementary Information:**

The online version contains supplementary material available at https://doi.org/10.1186/s12890-026-04519-1.

## Introduction

Idiopathic Pulmonary Fibrosis (IPF) is a rare, chronic, progressive and fibrotic interstitial lung disease with unclear etiology, affecting approximately 3 million people worldwide [1]. With global population aging, the incidence and prevalence of IPF are increasing, which are estimated at 0.09–1.30 and 0.33–4.51 per 10,000 persons, respectively [2]. Geographic variations in incidence are evident, with rates highest in North America and Europe, ranging from 3 to 9 cases per 100,000 person-years [3]. In contrast, incidence is lower in South America and East Asia, lower than 4 cases per 100,000 person-years, while in South Korea, it has risen from 3.56 to 7.91 cases per 100,000 person-years annually. [4]. IPF progresses rapidly, with a 1-year progression rate of 47.2% [5]. The prognosis is poor, with median survival after diagnosis of 2–3 years [6]. Despite extensive research, the etiology of IPF remains undefined, and therapeutic options remain limited. A thorough understanding of IPF pathogenesis is essential for developing effective early prevention and intervention strategies. Observational studies have identified several associated risk factors, including smoking, environmental exposure, and family history [7]. However, our understanding of modifiable risk factors is limited.

Umbrella reviews integrate existing meta-analyses to assess key factors including sample size, association strength, heterogeneity, and bias [8]. In recent years, umbrella reviews on IPF have focused on traditional Chinese medicine and pulmonary rehabilitation interventions. [9, 10]. Numerous potential risk factors for IPF, including environmental exposures [11], genetic factors [12], biomarkers [13], have not yet been systematically synthesized and analyzed. In addition to observational studies, Mendelian randomization (MR) studies are also used to explore potential causal risk factors. This statistical method uses genetic variations as instrumental variables to evaluate causal relationships between exposures and clinical outcomes. By using genetic variations as instrumental variables, confounding effects can be excluded, allowing more accurate causal inference [14]. MR studies are valuable for identifying modifiable factors that may help reduce IPF risk through interventions. Although MR studies have been widely applied to investigate potential causal risk factors for IPF [15, 16], existing meta-analyses and MR studies have yielded inconsistent and heterogeneous results, lacking comprehensive and unified evidence evaluation. Umbrella reviews can systematically integrate evidence from existing meta-analyses and MR studies, assess its credibility, and identify biases.

Current meta-analyses on IPF risk primarily include observational studies, which cannot establish definitive causal inferences due to inherent design limitations. In contrast, MR studies have unique advantages in causal inference, thus overcoming some limitations of traditional observational studies. Given this inconsistency, it is necessary to conduct an umbrella review to synthesize available evidence, and further assess the consistency and credibility of relevant research findings. This umbrella review follows the PECO framework to formulate the research question: P (Population, general adult population), E (Exposure, genetic, environmental, lifestyle, and comorbid factors), C (Comparison, no or low exposure), O (Outcome, IPF risk). This study synthesizes causal evidence from meta-analyses and MR studies, compares the consistency or differences in the results, offering new insights for understanding the onset risks of IPF. Through this comprehensive analysis, we aim to provide a scientific basis for prevention and early intervention strategies, thereby guiding future clinical practice of IPF.

## Methods

### Search strategy

We followed the Preferred Reporting Items for Systematic Reviews and Meta-Analyses (PRISMA) 2020 statement [17] for this study. The detailed checklis are provided in Supplementary Table 1. This study systematically searched eight databases, including PubMed, Embase, Cochrane Library, Web of Science, China National Knowledge Infrastructure (CNKI), Chongqing VIP (CQVIP), Wanfang Data, and China Biology Medicine (CBM), to identify systematic reviews and meta-analyses as well as MR studies concerning the risk factors for IPF published up to March 11, 2025. All databases were accessed through the institutional subscription provided by our affiliated hospital. Combined medical subject headings (MeSH) and free-text terms were used to tailor specific search strategies for each database. The search strategy involved key terms such as “idiopathic pulmonary fibrosis” “risk factors” “meta-analysis”, and “mendelian randomization.” The specific search strategies are provided in Supplementary Tables 2–3.

### Inclusion and exclusion criteria

#### Systematic review and meta-analysis

Inclusion criteria: (I) Systematic review and meta-analysis of IPF risk factors; (II) Effect estimates using odds ratios (OR) or hazard ratios (HR).

Exclusion criteria: (I) Systematic reviews without data synthesis or insufficient data for effect calculation; (II) Studies published in languages other than English; (III) Outdated, duplicate, or incomplete studies, conference abstracts, and studies with unavailable data.

### MR study

Inclusion criteria: (I) Any study using MR to explore the causal relationship between relevant factors and IPF risk, with positive results; (II) Studies using OR or HR for effect estimation, with inverse variance weighting (IVW) as the main method.

Exclusion criteria: (I) Studies unrelated to IPF risk, including magnetic resonance imaging and other imaging studies; (II) Non-English publications; (III) Duplicate publications, conference abstracts, and studies with incomplete or inaccessible data.

### Study selection

All data were imported into Endnote X8 software, and duplicates were removed. Siyuan Lei and Jun Wang independently screend the studies by titles and abstracts, then the eligible studies were finally determined by reading and evaluating the full text. Any disagreements were resolved by a third researcher to reach a consensus.

### Quality assessment

A Measure Tool to Assess Systematic Reviews (AMSTAR-2) is a tool used to assess the methodological quality of systematic review and meta-analysis [18]. It consists of 16 items, covering study design, literature screening, data extraction, and risk of bias assessment. Each item can be rated as “yes” (1 point), “partially” (0.5 point), or “no” (0 point). AMSTAR-2 particularly emphasizes methodological flaws in critical items, including 7 key items. The quality level is determined by compliance with key items and the number of defects in non-key items, and is divided into four levels: high, moderate, low, and critically low.

We used the Strengthening the Reporting of Observational Studies in Epidemiology-Mendelian Randomization (STROBE-MR) guidelines to evaluate the reporting quality of the included studies [19]. The checklist consists of six sections: title and abstract, introduction, methods, results, discussion, and other information, with a total of 20 items. Each item is defined as “yes” or “no”, depending on whether the authors have clearly stated it, and is scored as “1” or “0”, respectively. The quality assessment score is then converted into a percentage, with scores below 75%, between 75% and 85%, and above 85% being classified as high, moderate, and low bias risk, respectively [20]. Siyuan Lei and Jun Wang independently assessed meta-analyses using AMSTAR-2 and original MR studies using STROBE-MR, with disagreements resolved through team consensus. For detailed information on the checklist items, please refer to Supplementary Table 4.

### Data extraction

Huanrong Ruan and Hulei Zhao independently extracted key information using a pre-designed data extraction form. For systematic reviews and meta-analyses, the extracted information included title, first author, publication year, number of cases, exposures, effect size estimates, and quality assessment tools. Regarding MR studies, detailed information including the title, first author, publication year, ethnicity, exposures, database sources, causal effect estimates with 95% confidence intervals (CIs), sample size, and number of single-nucleotide polymorphisms (SNPs). Any disagreements were resolved by an independent third-party reviewer.

### Evidence grading criteria for associations from meta-analyses and MR studies

This umbrella review used a conceptual framework that combines associative evidence from meta-analyses with causal evidence from MR studies to guide evidence synthesis and result interpretation. Using this framework, we classified the credibility of evidence significantly associated with IPF into four levels: convincing, highly suggestive, suggestive, and weak. The assessment is based on statistical significance, sample size, 95% prediction interval (PI), heterogeneity, and publication bias, with reference to existing umbrella reviews [21–23]. We adopted the causal evidence grading criteria from the recent MR review by Markozannes et al. [24], which divides evidence into four levels: robust, probable, suggestive, and insufficient. The grading is based on the statistical significance of *P*-values and the consistency of the direction of effects.

### Statistical analysis

When there are multiple meta-analyses published at different times for the same exposure factor, we selected the latest or updated one with more included studies and a larger sample size as the best available evidence. Each IPF risk factor from eligible meta-analyses was regarded as an independent exposure-outcome association and counted separately, with effect sizes summarized by OR or HR. For MR studies, each exposure factor with a causal effect to IPF risk was defined as one independent association. When at least two independent studies reported a causal effect of an exposure factor or genetic instrument on IPF, we performed a meta-analysis using the metan command in STATA 15.1 [25], with pooled results counted as a single association to avoid duplicate counting. The primary outcome was the association between genetic variation and IPF risk, reported as OR with 95% CIs. Heterogeneity was assessed via Cochran’s Q test and I² statistic; I² > 50% indicated significant heterogeneity. Given the small number of included studies, Cochran’s Q test has limited statistical power, so the heterogeneity results should be interpreted with caution. When significant heterogeneity existed, we adopted a random effects model; otherwise, we used a fixed effects model. In this study, the results were considered statistically significant when the *P*<0.05.

## Results

### Study search and selection

**Fig. 1:**
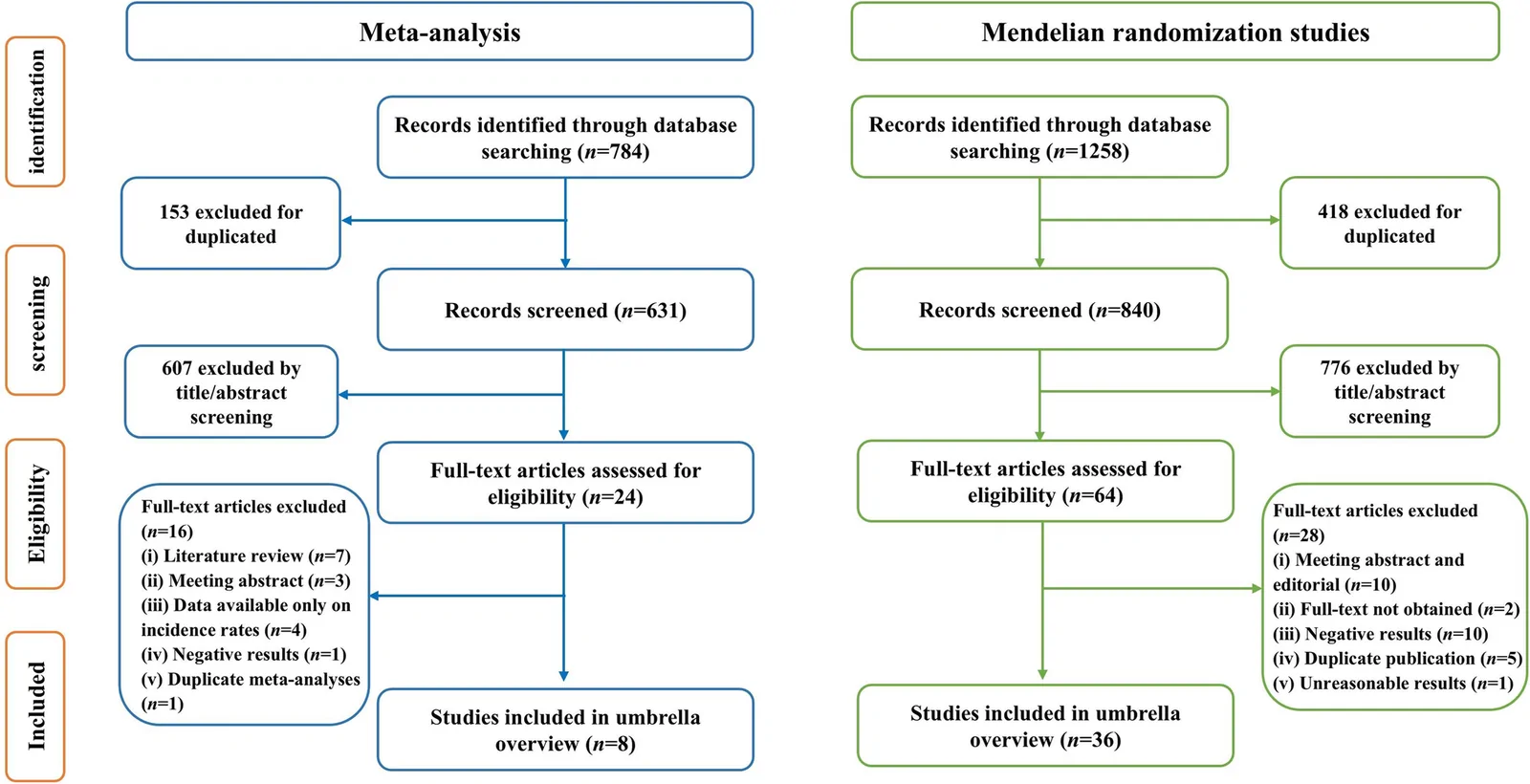
Flow diagram of study selection

A total of 784 records were initially retrieved for meta-analyses. After removing duplicates and screening by titles, abstracts and full texts, 8 meta-analyses were finally included. For MR studies, 1258 records were collected, and 36 MR studies were ultimately included after sequential screening. The detailed screening process is presented in Fig. 1. The list of excluded studies and corresponding reasons are provided in Supplementary Tables 5–6.

### Characteristics of included meta-analyses and summary on evidence grading

A total of 8 meta-analyses were included, reporting 22 associations, which were classified into 5 main categories: genetic factors [26] (*N* = 3), viral infection [27] (*N* = 5), occupational and environmental exposures [28–30] (*N* = 6), lifestyle [28, 31] (*N* = 2), and clinical variables [30, 32, 33] (*N* = 6). The total number of cases ranged from 674 to 424,849, with one study [31] not reporting the cases. The methodological quality assessment tools used by the meta-analyses were as follows. 6 studies used the Newcastle-Ottawa Scale (NOS), one [26] used the Strengthening the Reporting of Genetic Association Studies (STREGA), and one [28] used the Risk Of Bias Assessment in Non-randomized Studies of Interventions (RoBANS). The basic characteristics are shown in Supplementary Table 7.

The total AMSTAR-2 scores ranged from 6.5 to 13, with a median of 12.5. The critical items scores ranged from 2 to 6, with a median of 4.75. Among the 7 critical items, only one study [32] declared pre-specified methods, accounting for 12.5%. All studies conducted a comprehensive literature search. Only 2 studies [27, 29]. partially explained the excluded literature without a list, accounting for 12.5%. A total of 75% of studies considered risk of bias in result interpretation, and 62.5% discussed the impact of bias from individual studies. The quality of included meta-analyses was rated as low and critically low. Detailed AMSTAR-2 assessment for each study is shown in Supplementary Table 8.

According to the evidence grading criteria, 22 significant associations were graded, with detailed information grading shown in Supplementary Table 9. The pooled *P* values ranged from 0.001 to 0.050 (*N* = 22), with only 2 associations having *P* < 0.0001. Twelve associations included more than 1000 cases, and all excluded the null hypothesis. Eight associations reported significant heterogeneity with I² > 50%, 3 showed publication bias by Egger’s test, 9 had no significant publication bias, and 10 were not suitable for testing due to the limited studies. Overall, 12 associations were graded as suggestive and 10 as weak, mainly due to small sample size, large heterogeneity, and lack of publication bias testing.

### Results of meta-analyses in categories

**Fig. 2:**
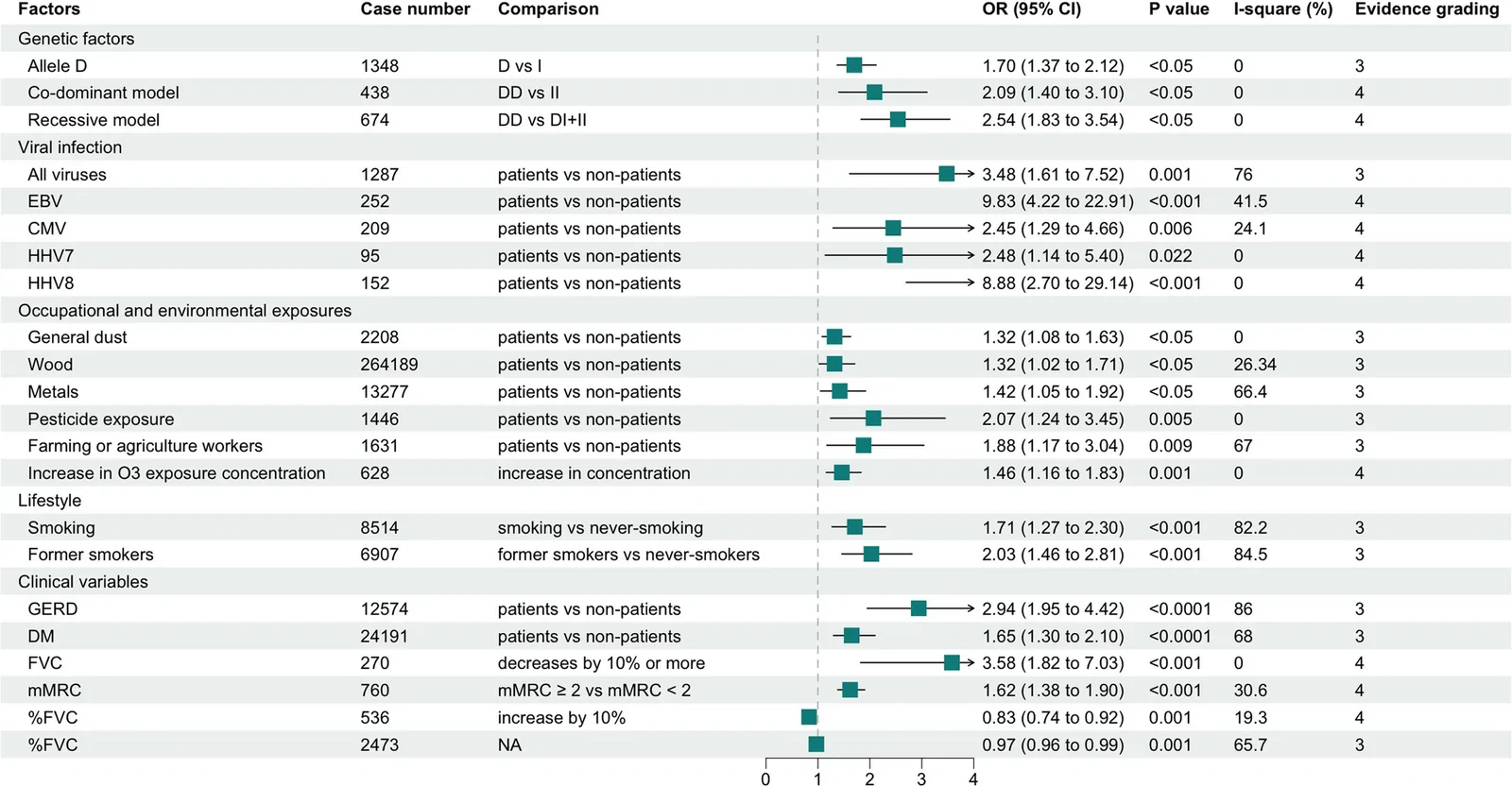
Forest plot of evidence grading for significant associations with the risk of IPF from meta-analyses

Three genetic factors increased IPF risk, which are the allele D (D vs. I, OR = 1.70), co-dominant model (DD vs. II, OR = 2.09), and recessive model (DD vs. DI + II, OR = 2.54). Five viral infections were associated with increased IPF risk. Only all viruses (OR = 3.48) showed suggestive evidence, while Epstein-Barr virus (EBV) (OR = 9.83), CytoMegalo virus (CMV) (OR = 2.45), Human herpesvirus 7 (HHV7) (OR = 2.48), and Human herpesvirus 8 (HHV8) (OR = 8.88) were all weak associations. Six occupational and environmental exposure factors were identified. General dust (OR = 1.32), wood (OR = 1.32), metals (OR = 1.42), pesticide exposure (OR = 2.07), and farming workers (OR = 1.88) all provided suggestive evidence. O3 exposure (OR = 1.46) was a risk factor for acute exacerbation of IPF, with weak association evidence. In lifestyle factors, only smoking increased IPF risk. Both current smoking (OR = 1.71), and former smoking (OR = 2.03) provided suggestive evidence. Six clinical variables were identified. Gastroesophageal reflux disease (GERD) (OR = 2.94) and diabetes mellitus (OR = 1.65) increase the risk of IPF, with evidence being suggestive. A decrease in Forced Vital Capacity (FVC) by 10% or more (HR = 3.58) and a modified Medical Research Council (mMRC) score of ≥ 2 (HR = 1.62) was identified as risk factors for IPF exacerbation, both showing weak associations. However, a 10% increase in %FVC (HR = 0.83) and %FVC (HR = 0.97) were identified as protective factors against IPF exacerbation, with weak and suggestive evidence, respectively. The evidence grading for significant associations with IPF risk from meta-analyses is shown in Fig. 2.

### Characteristics of included MR studies and summary on evidence grading

Thirty-six MR studies were published in 2023 and 2024. IPF datasets were derived from multiple sources, mainly including FinnGen, meta-analysis, genome-wide association study (GWAS), all involving European populations. The number of SNPs ranged from 3 to 543 and the sample size ranged from 186,176 to 635,172. The main analysis method was IVW. The basic features are presented in Supplementary Table 10.

STROBE-MR assessment indicated high reporting quality and low bias risk. Reporting quality results are summarized in Supplementary Table 11. A total of 124 associations were investigated. After merging, 108 associations remained, which were categorized as lifestyle [34–36] (*N* = 5), diet and nutrition [37, 38] (*N* = 6), anthropometric indices [39–42] (*N* = 6), biomarkers [35, 41–60] (*N* = 86), clinical variables, diseases, and treatments [40, 61–69] (*N* = 5) (Supplementary Table 10). Causal evidence grading for MR studies yielded 13 robust, 90 possible, 17 suggestive, and 4 insufficient associations.

### Results of MR studies in categories

Five lifestyle factors were identified. Age of smoking initiation (OR = 0.11) and maternal smoking (OR = 13.18) were found to have a positive association with the risk of IPF, with suggestive evidence. Smoking initiation (OR = 1.28) and lifetime smoking (OR = 1.62) were also likely to increase the risk of IPF, and both were assigned probable evidence. Moderate-to-vigorous physical activity (OR = 0.27) was likely to reduce the risk, with probable evidence.

Among the 124 associations, 6 dietary and nutritional factors were involved. Positive causal associations with IPF risk were observed for pork intake, average weekly beer plus cider intake, beef intake, and alcohol-related indicators, with evidence graded as probable, suggestive, robust, and suggestive, respectively. Negative causal associations were found for oily fish and yogurt intake, with suggestive and robust evidence, respectively.

A total of 6 anthropometric indices were identified. Among them, body mass index (BMI) was reported in four studies, with two rated as probable, one as suggestive and one as insufficient evidence. Waist circumference (WC) was addressed in two studies with probable evidence. Body fat percentage was examined in two studies, with one robust and one probable. All indices showed significant positive causal associations with IPF. The pooled results for BMI (OR = 1.45), WC (OR = 1.59), and body fat percentage (OR = 1.61) still indicate that they were risk factors for IPF (Supplementary Figs. 1–3). Other factors with positive causal associations included weight (OR = 1.44), whole body fat mass (OR = 1.40), and trunk fat mass (OR = 1.35), all considered probable evidence.

A total of 86 biomarkers were included. Three studies have demonstrated a causal association between telomere length and IPF. One study showed a positive association with insufficient evidence, while the other two showed a negative association with robust evidence. The combined telomere length (OR = 0.91) did not show a significant causal association (Supplementary Fig. 4). For gut microbiota, Family XIII and Ruminococcus gnavus were examined in three studies. One study reported a positive association, while two reported negative associations, all with probable evidence. After combining the results, no causal association was found (OR = 0.85; OR = 0.92) (Supplementary Figs. 5–6). Additionally, we also found robust evidence that CD8 on Terminally Differentiated CD8 + T cells (OR = 1.124) increases the risk of IPF. In contrast, several factors showed robust evidence of decreasing the risk of IPF, including fucosyltransferase 3 (FUT3) (OR = 0.81), Seropositive rheumatoid arthritis (RA) (OR = 0.93), and Glutamine levels (OR = 0.75). There are 66 associations with probable evidence. Among them, 30 biomarkers related to inflammatory markers, gut microbiota, and immune cells, such as interleukin-1 (IL-1), circulating C-reactive protein (CRP) levels, and herpes simplex virus infection showed an increased risk of IPF. Thirty-six biomarkers such as Adiponectin, Testosterone, Lipid-Carnitine, showed probable evidence of reducing the risk of IPF. Additionally, 11 biomarkers were identified as having suggestive causal association evidence.

**Fig. 3:**
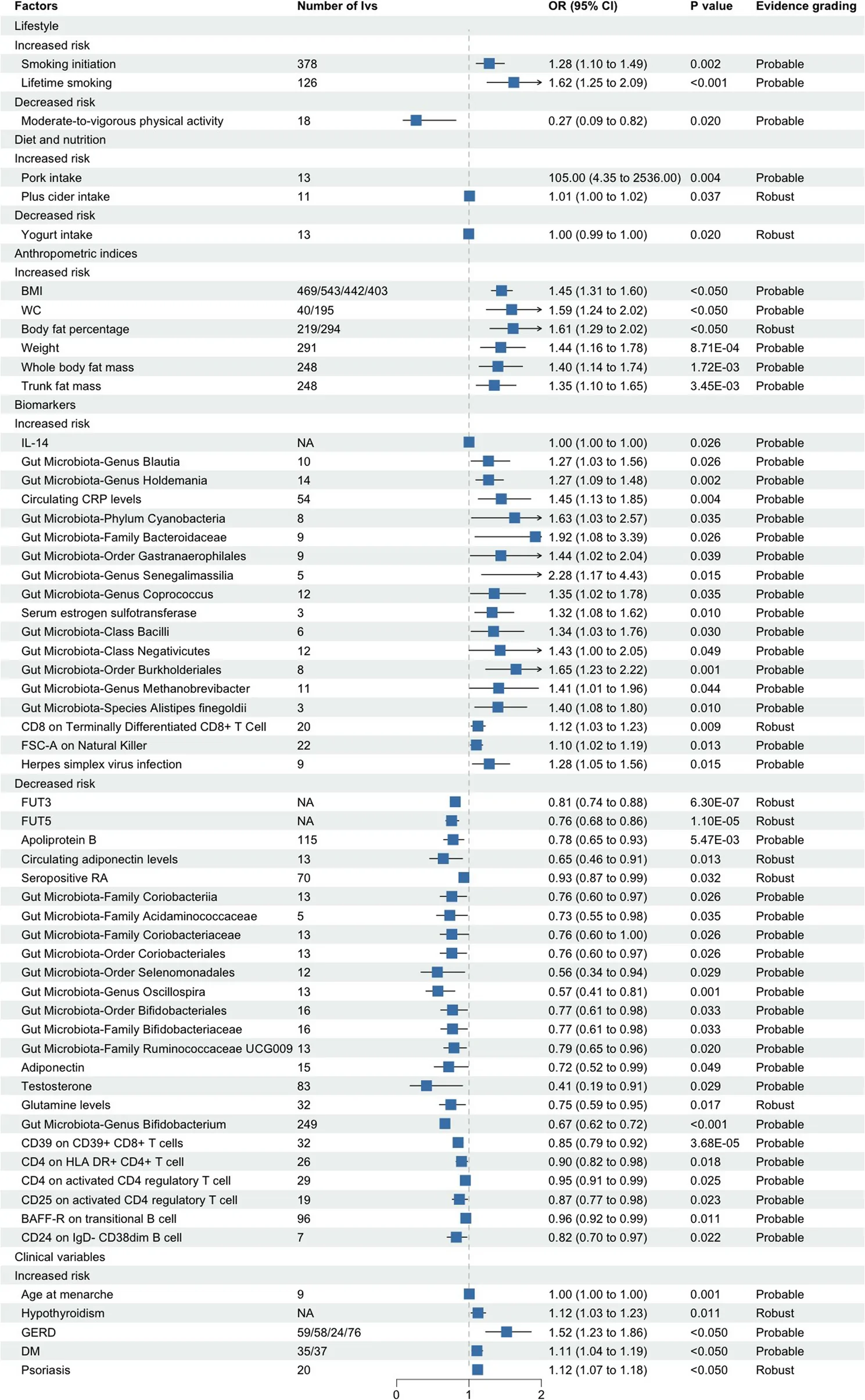
Forest plot of evidence grading for significant associations with the risk of IPF from MR studies

We identified 5 clinical variables that showed positive causal associations with IPF. GERD was reported in four studies, with evidence rated as two probable and two insufficient. Diabetes mellitus was addressed in two studies, both with probable evidence. Psoriasis was reported in two studies, with one showing robust evidence and one probable. The combined results for GERD (OR = 1.52), diabetes mellitus (OR = 1.11), and psoriasis (OR = 1.12) suggest an increased risk of IPF (Supplementary Figs. 7–9). Hypothyroidism (OR = 1.125) also showed robust evidence of increased risk. Age at menarche (OR = 1.004) indicated probable evidence of increased risk of IPF. Due to the limitations of the figures, the partial evidence grading for significant associations with IPF risk from MR studies is shown in Fig. 3.

### Comparison between associations derived from meta-analyses and MR studies

A total of three overlapping factors were identified by both meta-analysis and MR studies, all showing consistent significant associations, but no strong evidence was presented. Regarding smoking, meta-analysis provided suggestive evidence (smoking vs. never-smoking, OR = 1.71). Meanwhile, MR studies found probable evidence for smoking initiation (OR = 1.28), and for lifetime smoking (OR = 1.62). GERD showed suggestive evidence from meta-analysis (OR = 2.94). and the pooled MR result (OR = 1.52). For diabetes, meta-analysis provided suggestive evidence with an OR of 1.65, while the pooled MR result showed an OR of 1.11.

## Discussion

This umbrella review identified several risk factors associated with IPF, with smoking, GERD, and diabetes being the only factors consistently supported by both meta-analyses and MR studies, although robust evidence remains limited. Among 124 associations from MR studies, 13 were graded as robust and 90 as probable, whereas meta-analyses yielded 22 significant factors, all of which were classified as suggestive or weak evidence based on grading criteria. These findings highlight a substantial discrepancy in evidence strength between meta-analyses and MR studies, underscoring the need for more rigorous causal inference approaches in IPF risk factor research. This aligns with a large-scale appraisal by Ioannidis et al. [70], which demonstrated that statistically significant results are common in observational meta-analyses but rarely convincing when compared against MR evidence. Notably, this discrepancy contrasts with previous umbrella reviews based solely on observational studies, which often reported more “highly suggestive” evidence, suggesting that traditional observational studies may overestimate effect sizes due to residual confounding and reverse causation. Clinically, physicians should prioritize smoking, GERD, and diabetes as the three risk factors with causal evidence, while interpreting other factors from observational studies with caution. From a public health perspective, smoking control, GERD management, and diabetes care should be prioritized in preventive strategies for high-risk IPF populations. To our knowledge, this is the first umbrella review to synthesize evidence from both meta-analyses and MR studies on IPF risk factors, encompassing 8 meta-analyses and 36 MR studies covering genetic factors, viral infections, occupational and environmental exposures, lifestyle, and clinical variables. MR studies demonstrated good reporting quality based on STROBE-MR. This review provides a stronger evidence base for understanding IPF risk and guiding clinical and public health strategies.

### Genetic factors

Genetic factors play a key role in IPF pathogenesis, although their exact mechanisms remain unclear. Some studies have reported that certain insertion/deletion (I/D) polymorphisms in the introns of the angiotensin-converting enzyme (ACE) gene can affect the onset and progression of IPF [71]. Our meta-analysis identified three ACE gene factors, namely allele D, co-dominant model, and recessive model are associated with IPF risk. These factors may act by regulating vascular permeability, causing alveolar endothelial dysfunction, and promoting fibroblast proliferation [72]. The angiotensin II produced by their catalysis may promote cell regeneration and extracellular matrix deposition, contributing to pulmonary fibrosis [73]. However, the evidence was weak and not supported by MR analyses, indicating that the causal role of ACE polymorphisms in IPF remains to be clarified.

### Viral infection

IPF has long been linked to viral and bacterial infections. A previous meta-analysis reported pooled prevalence of 53.72% for viral and 31.21% for bacterial infections [74]. Our umbrella review similarly found that all viruses, EBV, CMV, HHV7, and HHV8 are associated with increased IPF risk. Yan et al. [75] provide preliminary evidence that EBV, CMV, and HSV, as well as herpesvirus-related IgG levels, are not causally linked to IPF. Herpesvirus infection may only be a phenomenon associated with pulmonary injury and fibrosis, and is considered to be related to vascular remodeling and pulmonary arterial hypertension in IPF [76]. Owing to the suggestive and weak evidence strength, the generalizability and stability of these findings remain limited.

### Occupational and environmental exposures

A 2019 survey found that exposure to silica, wood dust, metal dust, and agricultural dust increased the risk of IPF [77]. Our study further identified that general dust, wood, metals, pesticides, and farming or agricultural work may increase IPF risk, with suggestive evidence consistent with findings. Consistent with previous meta-analyses [78], metal dust, wood dust, pesticide, occupational history of farming or agriculture and ever smoking increased the risk of IPF. O3 exposure is considered a risk factor for acute IPF exacerbation, though evidence is weak. A recent meta-analysis further confirmed that O₃ and NO₂ exposure significantly increase the risk of IPF disease progression, supporting our findings [79].

### Lifestyle

Unfavorable lifestyle is closely associated with an increased risk of IPF [80]. Smoking is a key lifestyle risk factor for IPF, including former smoking, smoking initiation, lifetime smoking, and maternal smoking, all supported by suggestive evidence. A meta-analysis reported smoking is a risk factor for IPF and that former smoking is a poor prognostic factor for IPF [81]. We also found that earlier smoking initiation was associated with IPF risk, supported by probable MR evidence but not in meta-analyses. Moderate-to-vigorous physical activity may reduce IPF risk, supported by probable MR evidence. From a clinical and public health perspective, smoking cessation and regular physical activity represent promising modifiable strategies to reduce IPF risk and improve disease prognosis.

### Diet and nutrition

Our study identified 6 dietary factors associated with IPF susceptibility. Among them, pork, beer, alcohol, and beef showed positive causal associations. Pork may contribute to IPF development, while alcohol may increase risk by impairing lung repair, inducing inflammation, oxidative stress, or alveolar epithelial damage [82]. In contrast, oily fish, and yogurt were protective, with robust evidence for yogurt. Oily fish has been found to protect against lung inflammation and fibrosis in animal models [83]. Yogurt may regulate intestinal microbiota and influence respiratory immunity via the lung-gut axis [84]. Dietary modification represents a promising and modifiable preventive strategy to reduce IPF risk. Notably, these findings are derived from MR analyses and have not been confirmed by meta-analyses, indicating the need for further validation.

### Anthropometric indices

Low BMI and weight loss in the course of IPF may indicate a high risk of mortality in patients with IPF, so it is meaningful to monitor and manage the nutritional status of IPF patients [85]. While MR studies indicate genetic susceptibility to GERD increases IPF risk, this association weakens after BMI adjustment, suggesting that obesity reduction may be more effective than GERD intervention alone for IPF prevention [40]. Our study identified associations between obesity-related anthropometric indices, including both obesity degree and fat distribution, were associated with increased IPF risk. However, these associations lack confirmation in meta-analyses, highlighting the need for further research to determine whether modifying these indices could aid in IPF prevention.

### Biomarkers

This study reveals causal effects between 86 biomarkers and the risk of IPF. Immune cell-related biomarkers, specifically CD8 on terminally differentiated CD8 + T cells, have shown robust evidence of increasing the risk of IPF. Activation of IL-6 and IL-23 promotes IL-17 A production, which may trigger TGF-β and lead to pulmonary fibrosis [86, 87]. Chronic inflammation is a key characteristic of IPF, driven by both the innate and adaptive immune systems [88]. In this study, IL-1 and circulating CRP levels were associated with increased IPF risk. The UIP pattern is the primary pathological feature of IPF and may contribute to RA progression. We have found robust evidence that seropositive rheumatoid factor was associated with lower IPF risk. IPF may increase the risk of seropositivity, providing an additional basis for ILD assessment in RA patients [89, 90]. Disruption of the gut-lung axis may promote respiratory diseases, suggesting that gut microbiome as a potential disease modifier [91]. Specific microbiota, including actinomycetes, Streptococcus, and Holdemanella, were associated with increased IPF risk, possibly via inflammation and metabolic disorders [92]. These biomarker findings provide testable hypotheses for future studies and potential targets for early risk assessment, though clinical application requires further validation.

### Clinical variables

Gastric micro‑aspiration related to GERD may induce pulmonary inflammation and injury, increasing IPF risk [93]. This was consistent with our umbrella review, where the included meta-analyses provided suggestive evidence that GERD increases IPF risk. Furthermore, pooled MR analyses supported a significant positive causal association, strengthening the role of GERD in IPF pathogenesis. Although a potential association may exist between IPF and diabetes, the causal relationship between them remains unclear [94]. The meta-analysis and MR study included in our study both reached consistent conclusions, with the MR study further providing genetic evidence for a potential causal link between them. Additionally, MR studies provided robust evidence that hypothyroidism and psoriasis increase IPF risk, consistent with Sato et al. [95], supporting a link between thyroid function and pulmonary fibrosis. Thyroid hormones and antibodies may contribute to idiopathic interstitial pneumonia pathogenesis, possibly through altered plasma protein expression [96]. These findings highlight the importance of monitoring comorbidities in clinical practice to improve early identification and risk assessment of IPF.

### Limitations

Although this umbrella review identified associations between various factors and IPF risk, several limitations exist. First, the evidence strength for some associations is limited, particularly those graded as suggestive or probable, making them less reliable for clinical application. In addition, some included meta-analyses were of low quality, which may affect the reliability of the observational evidence. Second, although we tried to control for confounders, residual confounding could not be fully eliminated. Third, heterogeneity across studies due to disparities in region, ethnicity, enrollment criteria and detection methods may affect the consistency of results. Notably, all included MR studies were restricted to European populations, limiting the generalizability to other ethnicities, especially East Asian populations with increasing IPF incidence. Fourth, the preferential inclusion of MR studies with positive findings might introduce subtle selection bias, even after incorporating eligible borderline and non-significant results with strict quality control. Furthermore, potential issues such as winner’s curse and population stratification in MR analyses could influence the validity of causal inferences. Finally, we excluded non-English publications, which may have introduced language bias and limited the generalizability of our findings.

## Conclusions

This umbrella review systematically summarized evidence on IPF risk factors by integrating meta-analyses and MR studies. Meta-analyses identified 22 factors across genetics, infections, exposures, lifestyle, and clinical variables, but evidence credibility was generally low. MR studies revealed 108 associations across lifestyle, diet, anthropometrics, biomarkers, and clinical variables, with higher evidence levels. Smoking, GERD, and diabetes were the only factors consistently identified by both methods. Other factors could not be compared due to limited data. These findings support multifaceted prevention strategies for IPF but require cautious interpretation. Future large-scale prospective trials are needed to validate these results and inform early screening, prevention, and treatment.

### Recommendations

In clinical practice, clinicians should focus on three modifiable risk factors supported by causal evidence, including smoking cessation, GERD management and diabetes control. Caution is advised when interpreting other risk factors solely reported by observational studies before validation by genetic or experimental research. From a public health perspective, smoking control, GERD screening and treatment, and diabetes management should be prioritized in preventive strategies for high-risk IPF populations, and public health campaigns should raise awareness of these evidence-based risk factors among both the general population and healthcare providers. For future research, large-scale prospective studies and randomized controlled trials are needed to validate the causal relationships identified in this umbrella review. Further MR studies with larger sample sizes and more genetic instruments are warranted to explore risk factors that could not be adequately assessed due to limited data.

## Supplementary Information

Supplementary Material 1.

## Data Availability

All data generated or analyzed during the present study are included in this article and supplementary materials.
