# Supplementary material for: Risk factors for idiopathic pulmonary fibrosis: an umbrella review of meta-analyses and mendelian randomization analyses
Source: BMC Pulm Med. 2026 Jul 22;26:434. doi: 10.1186/s12890-026-04519-1 (PMC13617888; doi:10.1186/s12890-026-04519-1)
Supplement: Supplementary file 1 [file 12890_2026_4519_MOESM1_ESM.docx]

Supplementary Material

**1 Supplementary Tables**

**Table 1 PRISMA 2020 Checklist**

| **Section and Topic** | **Item #** | **Checklist item** | **Location where item is reported** |
| --- | --- | --- | --- |
| **TITLE** | | |  |
| Title | 1 | Identify the report as a systematic review. | P1 |
| **ABSTRACT** | | |  |
| Abstract | 2 | See the PRISMA 2020 for Abstracts checklist. | P2-3 |
| **INTRODUCTION** | | |  |
| Rationale | 3 | Describe the rationale for the review in the context of existing knowledge. | P3-5 |
| Objectives | 4 | Provide an explicit statement of the objective(s) or question(s) the review addresses. | P5 |
| **METHODS** | | |  |
| Eligibility criteria | 5 | Specify the inclusion and exclusion criteria for the review and how studies were grouped for the syntheses. | P5-6 |
| Information sources | 6 | Specify all databases, registers, websites, organisations, reference lists and other sources searched or consulted to identify studies. Specify the date when each source was last searched or consulted. | P5 |
| Search strategy | 7 | Present the full search strategies for all databases, registers and websites, including any filters and limits used. | P5 |
| Selection process | 8 | Specify the methods used to decide whether a study met the inclusion criteria of the review, including how many reviewers screened each record and each report retrieved, whether they worked independently, and if applicable, details of automation tools used in the process. | P6 |
| Data collection process | 9 | Specify the methods used to collect data from reports, including how many reviewers collected data from each report, whether they worked independently, any processes for obtaining or confirming data from study investigators, and if applicable, details of automation tools used in the process. | P7-8 |
| Data items | 10a | List and define all outcomes for which data were sought. Specify whether all results that were compatible with each outcome domain in each study were sought (e.g. for all measures, time points, analyses), and if not, the methods used to decide which results to collect. | P7-8 |
|  | 10b | List and define all other variables for which data were sought (e.g. participant and intervention characteristics, funding sources). Describe any assumptions made about any missing or unclear information. | P7-8 |
| Study risk of bias assessment | 11 | Specify the methods used to assess risk of bias in the included studies, including details of the tool(s) used, how many reviewers assessed each study and whether they worked independently, and if applicable, details of automation tools used in the process. | P6-7 |
| Effect measures | 12 | Specify for each outcome the effect measure(s) (e.g. risk ratio, mean difference) used in the synthesis or presentation of results. | P7 |
| Synthesis methods | 13a | Describe the processes used to decide which studies were eligible for each synthesis (e.g. tabulating the study intervention characteristics and comparing against the planned groups for each synthesis (item #5)). | P8-9 |
|  | 13b | Describe any methods required to prepare the data for presentation or synthesis, such as handling of missing summary statistics, or data conversions. | P8-9 |
|  | 13c | Describe any methods used to tabulate or visually display results of individual studies and syntheses. | P8-9 |
|  | 13d | Describe any methods used to synthesize results and provide a rationale for the choice(s). If meta-analysis was performed, describe the model(s), method(s) to identify the presence and extent of statistical heterogeneity, and software package(s) used. | P8-9 |
|  | 13e | Describe any methods used to explore possible causes of heterogeneity among study results (e.g. subgroup analysis, meta-regression). | P8-9 |
|  | 13f | Describe any sensitivity analyses conducted to assess robustness of the synthesized results. | P8-9 |
| Reporting bias assessment | 14 | Describe any methods used to assess risk of bias due to missing results in a synthesis (arising from reporting biases). | P6-7 |
| Certainty assessment | 15 | Describe any methods used to assess certainty (or confidence) in the body of evidence for an outcome. | P8 |
| **RESULTS** | | |  |
| Study selection | 16a | Describe the results of the search and selection process, from the number of records identified in the search to the number of studies included in the review, ideally using a flow diagram. | P9 |
|  | 16b | Cite studies that might appear to meet the inclusion criteria, but which were excluded, and explain why they were excluded. | P9 |
| Study characteristics | 17 | Cite each included study and present its characteristics. | P9-10  P11-12 |
| Risk of bias in studies | 18 | Present assessments of risk of bias for each included study. | P10、P12 |
| Results of individual studies | 19 | For all outcomes, present, for each study: (a) summary statistics for each group (where appropriate) and (b) an effect estimate and its precision (e.g. confidence/credible interval), ideally using structured tables or plots. | P10-15 |
| Results of syntheses | 20a | For each synthesis, briefly summarise the characteristics and risk of bias among contributing studies. | P10-12 |
|  | 20b | Present results of all statistical syntheses conducted. If meta-analysis was done, present for each the summary estimate and its precision (e.g. confidence/credible interval) and measures of statistical heterogeneity. If comparing groups, describe the direction of the effect. | P10-15 |
|  | 20c | Present results of all investigations of possible causes of heterogeneity among study results. | P10-15 |
|  | 20d | Present results of all sensitivity analyses conducted to assess the robustness of the synthesized results. | P10-15 |
| Reporting biases | 21 | Present assessments of risk of bias due to missing results (arising from reporting biases) for each synthesis assessed. | P10-12 |
| Certainty of evidence | 22 | Present assessments of certainty (or confidence) in the body of evidence for each outcome assessed. | P10-15 |
| **DISCUSSION** | | |  |
| Discussion | 23a | Provide a general interpretation of the results in the context of other evidence. | P15-21 |
|  | 23b | Discuss any limitations of the evidence included in the review. | P20-21 |
|  | 23c | Discuss any limitations of the review processes used. | P20-21 |
|  | 23d | Discuss implications of the results for practice, policy, and future research. | P21 |
| **OTHER INFORMATION** | | |  |
| Registration and protocol | 24a | Provide registration information for the review, including register name and registration number, or state that the review was not registered. | P3 |
|  | 24b | Indicate where the review protocol can be accessed, or state that a protocol was not prepared. | P3 |
|  | 24c | Describe and explain any amendments to information provided at registration or in the protocol. | P3 |
| Support | 25 | Describe sources of financial or non-financial support for the review, and the role of the funders or sponsors in the review. | P22 |
| Competing interests | 26 | Declare any competing interests of review authors. | P22 |
| Availability of data, code and other materials | 27 | Report which of the following are publicly available and where they can be found: template data collection forms; data extracted from included studies; data used for all analyses; analytic code; any other materials used in the review. | P22 |

*From:*  Page MJ, McKenzie JE, Bossuyt PM, Boutron I, Hoffmann TC, Mulrow CD, et al. The PRISMA 2020 statement: an updated guideline for reporting systematic reviews. BMJ 2021;372:n71. doi: 10.1136/bmj.n71

**Table 2 Details of the sudy search strategy of meta-analysis.**

| **Search** | **Query** | **Items found** |
| --- | --- | --- |
| **PubMed** | | |
| #1 | ("Idiopathic Pulmonary Fibrosis"[MeSH Terms]) | 8903 |
| #2 | ((((((((((("Idiopathic Pulmonary Fibros*"[Title/Abstract]) OR ("Idiopathic Lung Fibrosis"[Title/Abstract])) OR ("Pulmonary Fibros*"[Title/Abstract])) OR ("Pulmonary Fibros*, Idiopathic"[Title/Abstract])) OR ("IPF"[Title/Abstract])) OR ("Fibrocystic Pulmonary Dysplasia*"[Title/Abstract])) OR ("Cryptogenic Fibrosing Alveoliti*"[Title/Abstract])) OR ("Fibrosing Alveoliti*, Cryptogenic"[Title/Abstract])) OR ("Dysplasia, Fibrocystic Pulmonary"[Title/Abstract])) OR ("Usual Interstitial Pneumonia*"[Title/Abstract])) OR ("Interstitial Pneumonia*, Usual"[Title/Abstract])) OR ("UIP"[Title/Abstract]) | 30371 |
| #3 | #1 OR #2 | 32005 |
| #4 | "Risk Factors "[MeSH Terms] OR " Protective Factors"[MeSH Terms] OR "Risk Assessment"[MeSH Terms] | 1229626 |
| #5 | (((("Risk Factor*"[Title/Abstract]) OR ("Protective Factor*"[Title/Abstract])) OR ("Risk Assessment*"[Title/Abstract])) OR ("Predictive Factor*"[Title/Abstract])) OR ("Predictor*"[Title/Abstract]) | 1441616 |
| #6 | #4 OR #5 | 2168270 |
| #7 | ("Meta-Analysis as Topic"[MeSH Terms]) | 30968 |
| #8 | (((("Meta-analys*"[Title/Abstract]) OR ("Meta Analysis"[Title/Abstract])) OR ("Systematic Review"[Title/Abstract])) OR ("Literature Review"[Title/Abstract])) OR ("Pooled Analys*"[Title/Abstract]) | 613385 |
| #9 | #7 OR #8 | 621978 |
| #10 | #3 AND #6 AND #9 | 106 |
| **Embase** | | |
| #1 | 'lung fibrosis'/exp OR 'fibrosing alveolitis'/exp | 105714 |
| #2 | 'lung fibrosis':ab,ti OR 'fibrosing alveolitis':ab,ti OR 'pulmonary fibrosis':ab,ti OR 'fibrosis, lung':ab,ti OR 'alveolitis, fibrosing':ab,ti OR 'idiopathic interstitial fibrosis':ab,ti OR 'lung parenchymal fibrosis':ab,ti OR 'diffuse interstitial fibrosis':ab,ti OR 'lung fibrosis, interstitial':ab,ti OR 'fibrosis pulmonis':ab,ti OR 'ipf':ab,ti | 53350 |
| #3 | #1 OR #2 | 115802 |
| #4 | 'risk factor'/exp OR 'risk assessment'/exp OR 'predictor variable'/exp | 2082603 |
| #5 | 'risk factor*':ab,ti OR 'protective factor*':ab,ti OR 'risk assessment*':ab,ti OR 'risk evaluation':ab,ti OR 'predictive factor*':ab,ti OR 'predictor*':ab,ti | 2062465 |
| #6 | #4 OR #5 | 3133406 |
| #7 | 'meta analysis'/exp OR 'systematic review'/exp | 625684 |
| #8 | 'meta analysis':ab,ti OR 'analysis, meta':ab,ti OR 'meta-analysis':ab,ti OR 'review, systematic':ab,ti OR 'systematic review':ab,ti | 545308 |
| #9 | #7 OR #8 | 735376 |
| #9 | #3 AND #6 AND #9 | 395 |
| **Cochrane Library** | | |
| #1 | MeSH descriptor: [Idiopathic Pulmonary Fibrosis] explode all trees | 615 |
| #2 | (“Idiopathic Pulmonary Fibros*”):ti,ab,kw OR (“Idiopathic Lung Fibrosis”):ti,ab,kw OR (“Pulmonary Fibros*”):ti,ab,kw OR (“Pulmonary Fibros*, Idiopathic”):ti,ab,kw OR (“IPF”):ti,ab,kw | 1470 |
| #3 | (“Fibrocystic Pulmonary Dysplasia*”):ti,ab,kw OR (“Cryptogenic Fibrosing Alveoliti*”):ti,ab,kw OR (“Fibrosing Alveoliti*, Cryptogenic”):ti,ab,kw OR (“Dysplasia, Fibrocystic Pulmonary”):ti,ab,kw OR (“Usual Interstitial Pneumonia*”):ti,ab,kw | 81 |
| #4 | (“Interstitial Pneumonia*, Usual”):ti,ab,kw OR (“UIP”):ti,ab,kw | 140 |
| #5 | #1 OR #2 OR #3 OR #4 | 1671 |
| #6 | MeSH descriptor: [Risk Factors] explode all trees | 38474 |
| #7 | MeSH descriptor: [Protective Factors] explode all trees | 287 |
| #8 | MeSH descriptor: [Risk Assessment] explode all trees | 13720 |
| #9 | (“Risk Factor*”):ti,ab,kw OR (“Protective Factor*”):ti,ab,kw OR (“Risk Assessment*”):ti,ab,kw OR (“Predictive Factor*”):ti,ab,kw OR (“Predictor*”):ti,ab,kw | 88372 |
| #10 | #6 OR #7 OR #8 OR #9 | 112091 |
| #11 | MeSH descriptor: [Meta-Analysis as Topic] explode all trees | 1627 |
| #12 | (“Meta-analys*”):ti,ab,kw OR (“Meta Analysis”):ti,ab,kw OR (“Systematic Review”):ti,ab,kw OR (“Literature Review”):ti,ab,kw OR (“Pooled Analys*”):ti,ab,kw | 29205 |
| #13 | #11 OR #12 | 29205 |
| #14 | #5 AND #10 AND #13 | 6 |
| [**Web of Science**](https://apps.webofknowledge.com/home.do?SID=6BQQjiiMCVa9MgFvRpC) **core collection** | | |
| #1 | “Pulmonary Fibros*” (Topic) or “Lung Fibros*” (Topic) or “IPF” (Topic) or “Fibrosing Alveolitis” (Topic) or “Fibrosis, Lung” (Topic) or “Alveolitis, Fibrosing” (Topic) or “Fibrosis Pulmonis” (Topic) or “Idiopathic Interstitial Fibrosis” (Topic) or “Usual Interstitial Pneumonia*” (Topic) or “Interstitial Pneumonia*, Usual” (Topic) or UIP” (Topic) or “Fibrocystic Pulmonary Dysplasia*” (Topic) or “Cryptogenic Fibrosing Alveoliti*” (Topic) or “Dysplasia, Fibrocystic Pulmonary” (Topic) or “Fibrosing Alveoliti*, Cryptogenic” (Topic) or “Lung Parenchymal Fibrosis” (Topic) or “Diffuse Interstitial Fibrosis” (Topic) | 50332 |
| #2 | “Risk Factor*” (Topic) or “Protective Factor*” (Topic) or “Risk Assessment*” (Topic) or “Predictive Factor*” (Topic) or “Predictor*” (Topic) or “Risk Evaluation” (Topic) | 2108577 |
| #3 | “Meta-analys*” (Topic) or “Meta Analysis” (Topic) or “Analysis, Meta” (Topic) or “Systematic Review” (Topic) or “Review, Systematic” (Topic) or “Literature Review” (Topic) or “Pooled Analys*” (Topic) | 778000 |
| #4 | #1 AND #2 AND #3 | 141 |
| **China National Knowledge Infrastructure** | | |
| #1 | SU=(特发性肺纤维化+特发性肺间质纤维化+IPF+肺纤维化+肺间质纤维化+弥漫性肺间质纤维化+纤维性肺泡炎) AND SU=(危险因素+风险因素+影响因素+相关因素+预测因子) AND SU=(系统评价+**Meta**+系统综述+荟萃分析) | 4 |
| **Wanfang database** | | |
| #1 | (主题:(特发性肺纤维化) or 主题:(特发性肺间质纤维化) or 主题:(IPF) or 主题:(肺纤维化) or 主题:(肺间质纤维化) or 主题:(弥漫性肺间质纤维化) or主题:(纤维性肺泡炎)) and (主题:(危险因素) or 主题:(风险因素) or 主题:(影响因素) or 主题:(相关因素) or 主题:(预测因子)) and (主题:(系统评价) or 主题:(Meta) or 主题:(系统综述) or 主题:(荟萃分析)) | 117 |
| **Vip citation database** | | |
| #1 | (M=特发性肺纤维化OR M=特发性肺间质纤维化OR M=IPF OR M=肺纤维化OR M=肺间质纤维化OR M=弥漫性肺间质纤维化OR M=纤维性肺泡炎) AND (M=危险因素OR M=风险因素OR M=影响因素OR M=相关因OR M=预测因子) AND (M=系统评价 OR M= Meta OR M=系统综述 OR M=荟萃分析) | 10 |
| **Chinese Biomedical Literature Database** | |  |
| #1 | (“特发性肺纤维化” [常用字段:智能] OR “特发性肺间质纤维化” [常用字段:智能] OR “IPF” [常用字段:智能] OR “肺纤维化” [常用字段:智能] OR “肺间质纤维化” [常用字段:智能] OR “弥漫性肺间质纤维化” [常用字段:智能] OR “纤维性肺泡炎”[常用字段:智能]) | 14426 |
| #2 | (“危险因素” [常用字段:智能] OR “风险因素” [常用字段:智能] OR “影响因素” [常用字段:智能] OR “相关因素” [常用字段:智能] OR “预测因子” [常用字段:智能]) | 581171 |
| #3 | (“系统评价” [常用字段:智能] OR “Meta” [常用字段:智能] OR “系统综述” [常用字段:智能] OR “荟萃分析” [常用字段:智能]) | 65875 |
| #4 | #1 AND #2 AND #3 | 5 |

**Table 3** **Details of the sudy search strategy of MR studies.**

| **Search** | **Query** | **Items found** |
| --- | --- | --- |
| **PubMed** | | |
| #1 | ("Idiopathic Pulmonary Fibrosis"[MeSH Terms]) | 8903 |
| #2 | ((((((((((("Idiopathic Pulmonary Fibros*"[Title/Abstract]) OR ("Idiopathic Lung Fibrosis"[Title/Abstract])) OR ("Pulmonary Fibros*"[Title/Abstract])) OR ("Pulmonary Fibros*, Idiopathic"[Title/Abstract])) OR ("IPF"[Title/Abstract])) OR ("Fibrocystic Pulmonary Dysplasia*"[Title/Abstract])) OR ("Cryptogenic Fibrosing Alveoliti*"[Title/Abstract])) OR ("Fibrosing Alveoliti*, Cryptogenic"[Title/Abstract])) OR ("Dysplasia, Fibrocystic Pulmonary"[Title/Abstract])) OR ("Usual Interstitial Pneumonia*"[Title/Abstract])) OR ("Interstitial Pneumonia*, Usual"[Title/Abstract])) OR ("UIP"[Title/Abstract]) | 30371 |
| #3 | #1 OR #2 | 32005 |
| #4 | ("Mendelian Randomization Analysis"[MeSH Terms]) | 7394 |
| #5 | (((((("Mendelian Randomization"[Title/Abstract]) OR ("Mendelian Randomisation"[Title/Abstract])) OR ("Analysis, Mendelian Randomization"[Title/Abstract])) OR ("Instrumental Variable"[Title/Abstract])) OR ("MR"[Title/Abstract])) OR ("Causal Inference"[Title/Abstract])) OR ("Causal Association"[Title/Abstract])) OR ("Causal Relationship"[Title/Abstract])) | 217815 |
| #6 | #4 OR #5 | 218036 |
| #7 | #3 AND #6 | 182 |
| **Embase** | | |
| #1 | 'lung fibrosis'/exp OR 'fibrosing alveolitis'/exp | 105714 |
| #2 | 'lung fibrosis':ab,ti OR 'fibrosing alveolitis':ab,ti OR 'pulmonary fibrosis':ab,ti OR 'fibrosis, lung':ab,ti OR 'alveolitis, fibrosing':ab,ti OR 'idiopathic interstitial fibrosis':ab,ti OR 'lung parenchymal fibrosis':ab,ti OR 'diffuse interstitial fibrosis':ab,ti OR 'lung fibrosis, interstitial':ab,ti OR 'fibrosis pulmonis':ab,ti OR 'ipf':ab,ti | 53350 |
| #3 | #1 OR #2 | 115802 |
| #4 | 'mendelian randomization analysis'/exp OR 'instrumental variable analysis'/exp | 16515 |
| #5 | 'mendelian randomization':ab,ti OR 'mendelian randomisation':ab,ti OR 'instrumental variable':ab,ti OR 'analysis, mendelian randomization':ab,ti OR 'mr':ab,ti OR 'causal Inference':ab,ti OR 'causal association':ab,ti OR 'causal relationship':ab,ti | 266931 |
| #6 | #4 OR #5 | 268899 |
| #7 | #3 AND #6 | 642 |
| **Cochrane Library** | | |
| #1 | MeSH descriptor: [Idiopathic Pulmonary Fibrosis] explode all trees | 615 |
| #2 | (“Idiopathic Pulmonary Fibros*”):ti,ab,kw OR (“Idiopathic Lung Fibrosis”):ti,ab,kw OR (“Pulmonary Fibros*”):ti,ab,kw OR (“Pulmonary Fibros*, Idiopathic”):ti,ab,kw OR (“IPF”):ti,ab,kw | 1470 |
| #3 | (“Fibrocystic Pulmonary Dysplasia*”):ti,ab,kw OR (“Cryptogenic Fibrosing Alveoliti*”):ti,ab,kw OR (“Fibrosing Alveoliti*, Cryptogenic”):ti,ab,kw OR (“Dysplasia, Fibrocystic Pulmonary”):ti,ab,kw OR (“Usual Interstitial Pneumonia*”):ti,ab,kw | 81 |
| #4 | (“Interstitial Pneumonia*, Usual”):ti,ab,kw OR (“UIP”):ti,ab,kw | 140 |
| #5 | #1 OR #2 OR #3 OR #4 | 1671 |
| #6 | MeSH descriptor: [Mendelian Randomization Analysis] explode all trees | 862 |
| #7 | (“Mendelian Randomization”):ti,ab,kw OR (“Mendelian Randomisation”):ti,ab,kw OR (“Analysis, Mendelian Randomization”):ti,ab,kw OR (“Instrumental Variable”):ti,ab,kw OR (“MR”):ti,ab,kw | 9664 |
| #8 | (“Causal Inference”):ti,ab,kw OR (“Causal Association”):ti,ab,kw OR (“Causal Relationship”):ti,ab,kw | 1902 |
| #9 | #6 OR #7 OR #8 | 11028 |
| #10 | #5 AND #9 | 4 |
| [**Web of Science**](https://apps.webofknowledge.com/home.do?SID=6BQQjiiMCVa9MgFvRpC) **core collection** | | |
| #1 | “Pulmonary Fibros*” (Topic) or “Lung Fibros*” (Topic) or “IPF” (Topic) or “Fibrosing Alveolitis” (Topic) or “Fibrosis, Lung” (Topic) or “Alveolitis, Fibrosing” (Topic) or “Fibrosis Pulmonis” (Topic) or “Idiopathic Interstitial Fibrosis” (Topic) or “Usual Interstitial Pneumonia*” (Topic) or “Interstitial Pneumonia*, Usual” (Topic) or UIP” (Topic) or “Fibrocystic Pulmonary Dysplasia*” (Topic) or “Cryptogenic Fibrosing Alveoliti*” (Topic) or “Dysplasia, Fibrocystic Pulmonary” (Topic) or “Fibrosing Alveoliti*, Cryptogenic” (Topic) or “Lung Parenchymal Fibrosis” (Topic) or “Diffuse Interstitial Fibrosis” (Topic) | 50332 |
| #2 | “Mendelian Randomization” (Topic) or “Mendelian randomisation” (Topic) or “Analysis, Mendelian Randomization” (Topic) or “Instrumental Variable” (Topic) or “MR” (Topic) or “Causal Inference” (Topic) or “Causal Association”(Topic) or “Causal Relationship”(Topic) | 310493 |
| #3 | #1 AND #2 | 255 |
| **China National Knowledge Infrastructure** | | |
| #1 | SU=(特发性肺纤维化+特发性肺间质纤维化+IPF+肺纤维化+肺间质纤维化+弥漫性肺间质纤维化+纤维性肺泡炎) AND SU=(孟德尔+工具变量+MR) | 12 |
| **Wanfang database** | | |
| #1 | (主题:(特发性肺纤维化) or 主题:(特发性肺间质纤维化) or 主题:(IPF) or 主题:(肺纤维化) or 主题:(肺间质纤维化) or 主题:(弥漫性肺间质纤维化) or主题:(纤维性肺泡炎)) and (主题:(孟德尔) or 主题:(工具变量) or 主题:(MR)) | 107 |
| **Vip citation database** | | |
| #1 | (M=特发性肺纤维化OR M=特发性肺间质纤维化OR M=IPF OR M=肺纤维化OR M=肺间质纤维化OR M=弥漫性肺间质纤维化OR M=纤维性肺泡炎) AND (M=孟德尔OR M=工具变量OR M=MR) | 21 |
| **Chinese Biomedical Literature Database** | |  |
| #1 | (“特发性肺纤维化” [常用字段:智能] OR “特发性肺间质纤维化” [常用字段:智能] OR “IPF” [常用字段:智能] OR “肺纤维化” [常用字段:智能] OR “肺间质纤维化” [常用字段:智能] OR “弥漫性肺间质纤维化” [常用字段:智能] OR “纤维性肺泡炎”[常用字段:智能]) | 14426 |
| #2 | (“孟德尔” [常用字段:智能] OR “工具变量” [常用字段:智能] OR “MR” [常用字段:智能]) | 35795 |
| #3 | #1 AND #2 | 35 |

**Table 4** **Detailed description of STROBE-MR checklist items.**

**STROBE-MR checklist of recommended items to address in reports of Mendelian randomization studies**

| **Item No** | **Section/Topic** |  | **Checklist Item** |
| --- | --- | --- | --- |
|  | **Title and Abstract** |  |  |
| 1 |  |  | Indicate Mendelian randomization (MR) as the study’s design in the title and/or the abstract if that is a main purpose of the study |
|  | **Introduction** |  |  |
| 2 | Background |  | Explain the scientific background and rationale for the reported study. What is the exposure? Is a potential causal relationship between exposure and outcome plausible? Justify why MR is a helpful method to address the study question |
| 3 | Objectives |  | State specific objectives clearly, including pre-specified causal hypotheses (if any). State that MR is a method that, under specific assumptions, intends to estimate causal effects |
|  | **Methods** |  |  |
| 4 | Study design and data sources |  | Present key elements of the study design early in the article. Consider including a table listing sources of data for all phases of the study. For each data source contributing to the analysis, describe the following: |
|  |  | a) | Setting: Describe the study design and the underlying population, if possible. Describe the setting, locations, and relevant dates, including periods of recruitment, exposure, follow-up, and data collection, when available. |
|  |  | b) | Participants: Give the eligibility criteria, and the sources and methods of selection of participants. Report the sample size, and whether any power or sample size calculations were carried out prior to the main analysis |
|  |  | c) | Describe measurement, quality control and selection of genetic variants |
|  |  | d) | For each exposure, outcome, and other relevant variables, describe methods of assessment and diagnostic criteria for diseases |
|  |  | e) | Provide details of ethics committee approval and participant informed consent, if relevant |
| 5 | Assumptions |  | Explicitly state the three core IV assumptions for the main analysis (relevance, independence and exclusion restriction) as well assumptions for any additional or sensitivity analysis |
| 6 | Statistical methods: main analysis |  | Describe statistical methods and statistics used |
|  |  | a) | Describe how quantitative variables were handled in the analyses (i.e., scale, units, model) |
|  |  | b) | Describe how genetic variants were handled in the analyses and, if applicable, how their weights were selected |
|  |  | c) | Describe the MR estimator (e.g. two-stage least squares, Wald ratio) and related statistics. Detail the included covariates and, in case of two-sample MR, whether the same covariate set was used for adjustment in the two samples |
|  |  | d) | Explain how missing data were addressed |
|  |  | e) | If applicable, indicate how multiple testing was addressed |
| 7 | Assessment of assumptions |  | Describe any methods or prior knowledge used to assess the assumptions or justify their validity |
| 8 | Sensitivity analyses and additional analyses |  | Describe any sensitivity analyses or additional analyses performed (e.g. comparison of effect estimates from different approaches, independent replication, bias analytic techniques, validation of instruments, simulations) |
| 9 | Software and pre-registration |  |  |
|  |  | a) | Name statistical software and package(s), including version and settings used |
|  |  | b) | State whether the study protocol and details were pre-registered (as well as when and where) |
|  | **Results** |  |  |
| 10 | Descriptive data |  |  |
|  |  | a) | Report the numbers of individuals at each stage of included studies and reasons for exclusion. Consider use of a flow diagram |
|  |  | b) | Report summary statistics for phenotypic exposure(s), outcome(s), and other relevant variables (e.g. means, SDs, proportions) |
|  |  | c) | If the data sources include meta-analyses of previous studies, provide the assessments of heterogeneity across these studies |
|  |  | d) | For two-sample MR: i. Provide justification of the similarity of the genetic variant-exposure associations between the exposure and outcome samples; ii. Provide information on the number of individuals who overlap between the exposure and outcome studies |
| 11 | Main results |  |  |
|  |  | a) | Report the associations between genetic variant and exposure, and between genetic variant and outcome, preferably on an interpretable scale |
|  |  | b) | Report MR estimates of the relationship between exposure and outcome, and the measures of uncertainty from the MR analysis, on an interpretable scale, such as odds ratio or relative risk per SD difference |
|  |  | c) | If relevant, consider translating estimates of relative risk into absolute risk for a meaningful time period |
|  |  | d) | Consider plots to visualize results (e.g. forest plot, scatterplot of associations between genetic variants and outcome versus between genetic variants and exposure) |
| 12 | Assessment of assumptions |  |  |
|  |  | a) | Report the assessment of the validity of the assumptions |
|  |  | b) | Report any additional statistics (e.g., assessments of heterogeneity across genetic variants, such as I^2^, Q statistic or E-value) |
|  |  |  |  |
| 13 | Sensitivity analyses and additional analyses |  |  |
|  |  | a) | Report any sensitivity analyses to assess the robustness of the main results to violations of the assumptions |
|  |  | b) | Report results from other sensitivity analyses or additional analyses |
|  |  | c) | Report any assessment of direction of causal relationship (e.g., bidirectional MR) |
|  |  | d) | When relevant, report and compare with estimates from non-MR analyses |
|  |  | e) | Consider additional plots to visualize results (e.g., leave-one-out analyses) |
|  | **Discussion** |  |  |
| 14 | Key results |  | Summarize key results with reference to study objectives |
| 15 | Limitations |  | Discuss limitations of the study, taking into account the validity of the IV assumptions, other sources of potential bias, and imprecision. Discuss both direction and magnitude of any potential bias and any efforts to address them |
| 16 | Interpretation |  |  |
|  |  | a) | Meaning: Give a cautious overall interpretation of results in the context of their limitations and in comparison with other studies |
|  |  | b) | Mechanism: Discuss underlying biological mechanisms that could drive a potential causal relationship between the investigated exposure and the outcome, and whether the gene-environment equivalence assumption is reasonable. Use causal language carefully, clarifying that IV estimates may provide causal effects only under certain assumptions |
|  |  | c) | Clinical relevance: Discuss whether the results have clinical or public policy relevance, and to what extent they inform effect sizes of possible interventions |
| 17 | Generalizability |  | Discuss the generalizability of the study results (a) to other populations, (b) across other exposure periods/timings, and (c) across other levels of exposure |
|  | **Other information** |  |  |
| 18 | Funding |  | Describe sources of funding and the role of funders in the present study and, if applicable, sources of funding for the databases and original study or studies on which the present study is based |
| 19 | Data and data sharing |  | Provide the data used to perform all analyses or report where and how the data can be accessed, and reference these sources in the article. Provide the statistical code needed to reproduce the results in the article, or report whether the code is publicly accessible and if so, where |
| 20 | Conflicts of Interest |  | All authors should declare all potential conflicts of interest |

Note: This checklist is copyrighted by the Equator Network under the Creative Commons Attribution 3.0 Unported (CC BY 3.0) license.

**Table 5 List of excluded meta-analyses with reasons for exclusion.**

| **Author, Year** | **Title** | **Reasons** |
| --- | --- | --- |
| Hershcovici, 2011 | Systematic review: the relationship between interstitial lung diseases and gastro-oesophageal reflux disease | Literature review (7) |
| Quah, E, 2018 | Systematic literature review investigating whether methotrexate causes chronic interstitial pulmonary fibrosis |  |
| Bano, 2020 | Thyroid Function and the Risk of Fibrosis of the Liver, Heart, and Lung in Humans: A Systematic Review and Meta-Analysis |  |
| Harari, 2020 | Fibrotic interstitial lung diseases and air pollution: a systematic literature review |  |
| Manna, 2021 | Environmental and occupational exposures and the risk of idiopathic pulmonary fibrosis: A systematic review |  |
| Wang, 2022 | Inflammatory bowel disease and risk of idiopathic pulmonary fibrosis: A protocol for systematic review and meta-analysis |  |
| Zamfir, 2023 | Exploring the Role of Biomarkers Associated with Alveolar Damage and Dysfunction in Idiopathic Pulmonary Fibrosis-A Systematic Review |  |
| Aljabban, 2019 | Elucidating idiopathic pulmonary fibrosis pathogenesis through meta-analysis of public data | Conference abstract (3) |
| Hassan, 2021 | A SYSTEMATIC REVIEW AND META-ANALYSIS OF GENETIC POLYMORPHISMS ASSOCIATED WITH THE RISK OF IDIOPATHIC PULMONARY FIBROSIS |  |
| Gandhi, 2023 | The Impact of Occupational Exposures on the Risk of Idiopathic Pulmonary Fibrosis - A Systematic Review and Meta-analysis |  |
| Kaunisto, 2013 | Idiopathic pulmonary fibrosis - a systematic review on methodology for the collection of epidemiological data | Data available only onincidence rates (4) |
| Han, 2015 | Diagnostic yield and postoperative mortality associated with surgical lung biopsy for evaluation of interstitial lung diseases: A systematic review and meta-analysis |  |
| Mostafaei, 2021 | The role of viral and bacterial infections in the pathogenesis of IPF: a systematic review and meta-analysis |  |
| Wang, 2024 | The incidence of acute exacerbation of idiopathic pulmonary fibrosis: a systematic review and meta-analysis |  |
| He, 2016 | Diabetes mellitus in idiopathic pulmonary fibrosis: A systematic review and meta-analysis | Duplicate meta-analyses (1) |
| Li, 2021 | Associations Between Diabetes and Idiopathic Pulmonary Fibrosis: a Study-level Pooled Analysis of 26 Million People | Negative results (1) |

**Table 6 List of excluded MR studies with reasons for exclusion.**

| **Author, Year** | **Title** | **Reasons** |
| --- | --- | --- |
| Cerani, 2019 | A metabolic biomarker for idiopathic pulmonary fibrosis: a two sample Mendelian randomization study and a case-control study | Meeting abstract and editorial  (10) |
| Duckworth, 2019 | Evidence that telomere length is causal for idiopathic pulmonary fibrosis but not chronic obstructive pulmonary disease: A UK Biobank Mendelian randomisation study |  |
| Reynolds, 2021 | Mendelian randomization study of cigarette smoking in idiopathic pulmonary fibrosis |  |
| Moss, 2023 | ASSESSING CAUSAL RELATIONSHIPS BETWEEN TYPE 2 DIABETES AND IDIOPATHIC PULMONARY FIBROSIS |  |
| Newton, 2023 | Gastro-oesophageal reflux and idiopathic pulmonary fibrosis: sorting the chicken and the egg by genetic link |  |
| Sangani, 2023 | Exploring the Causal Effects of Circulating Galectin-3 and sRAGE on Interstitial Lung Abnormalities Risk: A Mendelian Randomization Study |  |
| Guo, 2024 | The Causal Relationship Between Gut Microbiota and Idiopathic Pulmonary Fibrosis: A Two-sample Mendelian Randomization Study |  |
| Siranart, 2024 | Exploring the Relationship Between Body Mass Index, Smoking, and Idiopathic Pulmonary Fibrosis: Insights From a Mendelian Randomization Study |  |
| Zhang, 2024 | Genetic Association of Circulating Adipokines with Risk of Idiopathic Pulmonary Fibrosis: A Two-Sample Mendelian Randomization Study |  |
| 石钰洁, 2024 | 胃食管反流病与特发性纤维化间的因果关系：一项双向、两样本孟德尔随机化研究 |  |
| Yang, 2023 | Smoking increases the risk of idiopathic pulmonary fibrosis by reducing IGF1R levels: A Mendelian randomization and RNA-seq study | Full-text not obtained  (2) |
| Zhou, 2023 | Iron status and the risk of idiopathic pulmonary fibrosis: a mendelian randomization study |  |
| Lin, 2023 | Vitamin D and idiopathic pulmonary fibrosis: a two-sample mendelian randomization study | Negative results (10) |
| Sun, 2023 | Mendelian randomization analysis suggests no causal influence of gastroesophageal reflux disease on the susceptibility and prognosis of idiopathic pulmonary fibrosis |  |
| Yan, 2023 | Mendelian randomization reveals no correlations between herpesvirus infection and idiopathic pulmonary fibrosis |  |
| Zhang, 2023 | Using a Two-Sample Mendelian Randomization Study Based on Genome-Wide Association Studies to Assess and Demonstrate the Causal Effects of Allergic Rhinitis on Chronic Lower Respiratory Diseases and Lung Function |  |
| Zhou, 2023 | Causal relationship between lung diseases and risk of esophageal cancer: insights from Mendelian randomization |  |
| Hong, 2024 | Causal associations of obstructive sleep apnea with Chronic Respiratory Diseases: a Mendelian Randomization study |  |
| Lin, 2024 | Gastroesophageal reflux disease and idiopathic pulmonary fibrosis risk: A mendelian randomization study |  |
| Ma, 2024 | Causal Relationships Between Emotional Instability and Respiratory Diseases: A Mendelian Randomization Analysis |  |
| Moss, 2024 | Assessing Causal Relationships Between Diabetes Mellitus and Idiopathic Pulmonary Fibrosis |  |
| Wang, 2024 | The association of maternal smoking around birth with chronic respiratory diseases in adult offspring: A Mendelian randomization study |  |
| 张雅楠, 2022 | 甲状腺功能减退症与特发性肺纤维化因果关联的孟德尔随机化研究 | Duplicate publication (5) |
| Liu, 2023 | Immune-mediated inflammatory diseases and leukocyte telomere length: A Mendelian randomization study |  |
| Reynolds, 2023 | The causal relationship between gastro-oesophageal reflux disease and idiopathic pulmonary fibrosis: a bidirectional two-sample Mendelian randomisation study |  |
| Leavy, 2024 | Rheumatoid arthritis and idiopathic pulmonary fibrosis: a bidirectional Mendelian randomisation study |  |
| Wu, 2024 | Exploring genetic causal association between intestinal flora and idiopathic pulmonary fibrosis based on “gut-lung axis” and predicting intervention with traditional Chinese medicine |  |
| 冯悦, 2024 | 内脏脂肪组织与肺部疾病的孟德尔随机化研究 | Unreasonable results (1) |

**Table 7** **Basic characteristics of included meta-analyses and evidence grading results.**

| Author, year | Quality Assessment Tool | Factors | Number of IPF cases | Type of metric | Comparison | Summary of metric | *P* value | Heterogeneity (I^2^, %) | Egger’s P value | AMSTAR-2 total score | Evidence  grading |
| --- | --- | --- | --- | --- | --- | --- | --- | --- | --- | --- | --- |
| Chen et al. 2015 | STREGA | allele D | 1348 | OR | D vs I | 1.70 (1.37, 2.12) | <0.05 | 0 | NA | 6.5 | 3 |
|  |  | co-dominant model | 438 | OR | DD vs II | 2.09 (1.40, 3.10) | <0.05 | 0 | NA |  | 4 |
|  |  | recessive model | 674 | OR | DD vs DI+II | 2.54 (1.83, 3.54) | <0.05 | 0 | NA |  | 4 |
| Bédard Méthot et al. 2018 | NOS | GERD | 12574 | OR | patients vs non-patients | 2.94 (1.95, 4.42) | <0.0001 | 86 | NA | 12.5 | 3 |
| Sheng et al. 2020 | NOS | All viruses | 1287 | OR | patients vs non-patients | 3.48 (1.61, 7.52) | 0.001 | 76 | 0.001 | 13 | 3 |
|  |  | EBV | 252 | OR | patients vs non-patients | 9.83 (4.22, 22.91) | <0.001 | 41.5 | 0.03 |  | 4 |
|  |  | CMV | 209 | OR | patients vs non-patients | 2.45 (1.29, 4.66) | 0.006 | 24.1 | 0.355 |  | 4 |
|  |  | HHV7 | 95 | OR | patients vs non-patients | 2.48 (1.14, 5.40) | 0.022 | 0 | 0.702 |  | 4 |
|  |  | HHV8 | 152 | OR | patients vs non-patients | 8.88 (2.70, 29.14) | <0.001 | 0 | 0.508 |  | 4 |
| Bai et al. 2021 | NOS | Diabetes mellitus | 24191 | OR | patients vs non-patients | 1.65 (1.30, 2.10) | <0.0001 | 68 | 0.016 | 12.5 | 3 |
| Park et al. 2021 | RoBANS | metal dust | 2845 | OR | patients vs non-patients | 1.83 (1.15, 2.91) | 0.01 | 54 | NA | 11 | 3 |
|  |  | wood dust | 1599 | OR | patients vs non-patients | 1.62 (1.04, 2.53) | 0.03 | 5 | NA |  | 3 |
|  |  | pesticide exposure | 1446 | OR | patients vs non-patients | 2.07 (1.24, 3.45) | 0.005 | 0 | NA |  | 3 |

**Table 7 (Continued).**

| Author, year | Quality Assessment Tool | Factors | Number of IPF cases | Type of metric | Comparison | Summary of metric | P value | Heterogeneity (I^2^, %) | Egger’s P value | AMSTAR-2 total score | Evidence  grading |
| --- | --- | --- | --- | --- | --- | --- | --- | --- | --- | --- | --- |
|  |  | farming or agriculture workers | 1631 | OR | patients vs non-patients | 1.88 (1.17, 3.04) | 0.009 | 67 | NA |  | 3 |
|  |  | smoker | 807 | OR | patients vs non-patients | 1.38 (1.09, 1.74) | 0.008 | 10 | NA |  | 4 |
| Pauchet et al. 2022 | NOS | general dust | 2208 | OR | patients vs non-patients | 1.32 (1.08, 1.63) | <0.05 | 0 | ＞0.05 | 12.5 | 3 |
|  |  | wood | 264189 | OR | patients vs non-patients | 1.32 (1.02, 1.71) | <0.05 | 26.34 | ＞0.05 |  | 3 |
|  |  | metals | 13277 | OR | patients vs non-patients | 1.42 (1.05, 1.92) | <0.05 | 66.4 | 0.53 |  | 3 |
| Chen et al. 2023 | NOS | FVC | 270 | HR | decreases by 10% or more | 3.58 (1.82, 7.03) | <0.001 | 0 | NA | 12 | 4 |
|  |  | mMRC ≥ 2 | 760 | HR | mMRC ≥ 2 vs mMRC < 2 | 1.62 (1.38, 1.90) | <0.001 | 30.6 | NA |  | 4 |
|  |  | O3 exposure | 628 | HR | increase in concentration | 1.46 (1.16, 1.83) | 0.001 | 0 | NA |  | 4 |
|  |  | %FVC | 536 | HR | increase by 10% | 0.83 (0.74, 0.92) | 0.001 | 19.3 | NA |  | 4 |
|  |  | %FVC | 2473 | HR | NA | 0.97 (0.96, 0.99) | 0.001 | 65.7 | 0.118 |  | 3 |
| Fang et al. 2024 | NOS | smoking | 8514 | OR | smoking vs never-smoking | 1.71 (1.27, 2.30) | <0.001 | 82.2 | ＞0.05 | 13 | 3 |
|  |  | former smokers | 6907 | OR | former smokers vs never-smokers | 2.03 (1.46, 2.81) | <0.001 | 84.5 | ＞0.05 |  | 3 |

Abbreviations: STREGA: Strengthening the reporting of genetic association studies; NA: not available; NOS: Newcastle-Ottawa scale; GERD: Gastroesophageal reflux disease; EBV: Epstein-Barr virus; CMV: CytoMegalo virus; HHV7: Human herpesvirus 7; HHV8: Human herpesvirus 8; RoBANS: Risk of bias assessment in non-randomized studies; FVC: Forced vital capacity; mMRC: modified medical research council; OR: Odds ratio; HR: Hazard ratio.

**Table** **8 Details of AMSTAR-2 grading for quality of meta-analyses.**

| **Study** | **1** | **2^*^** | **3** | **4^*^** | **5** | **6** | **7^*^** | **8** | **9^*^** | **10** | **11^*^** | **12** | **13^*^** | **14** | **15^*^** | **16** | **critical item score** | **total score** | **Overall Confidence** |
| --- | --- | --- | --- | --- | --- | --- | --- | --- | --- | --- | --- | --- | --- | --- | --- | --- | --- | --- | --- |
| **Chen et al. 2015** | Y | N | Y | Y | N | Y | N | PY | N | N | Y | N | N | N | N | N | 2 | 5.5 | Critically low |
| **Bédard Méthot et al. 2019** | Y | Y | Y | Y | Y | Y | N | Y | Y | N | Y | Y | Y | PY | N | Y | 5 | 12.5 | Critically low |
| **Sheng et al. 2020** | Y | N | Y | Y | Y | Y | PY | Y | Y | N | Y | Y | Y | Y | Y | PY | 5.5 | 13 | Low |
| **Bai et al. 2021** | Y | N | Y | Y | Y | Y | N | Y | Y | N | Y | Y | Y | Y | PY | Y | 4.5 | 12.5 | Critically low |
| **Park et al. 2021** | Y | N | Y | Y | N | Y | N | Y | Y | N | Y | Y | PY | PY | Y | Y | 4.5 | 11 | Critically low |
| **Pauchet et al. 2022** | Y | N | Y | Y | Y | Y | PY | Y | Y | N | Y | Y | PY | Y | PY | Y | 4.5 | 12.5 | Low |
| **Chen et al. 2023** | Y | N | Y | Y | Y | Y | N | Y | Y | N | Y | Y | Y | Y | Y | N | 5 | 12 | Critically low |
| **Fang et al. 2024** | Y | Y | Y | Y | Y | Y | N | Y | Y | N | Y | Y | Y | Y | Y | Y | 5 | 13 | Critically low |

Abbreviations: Y: yes (1 point); PY: partial yes (0.5 point); N: no (0 point). * denoting the critical AMSTAR-2 items; critical item score= total score of 0 (N), 0.5 (PY), and 1 (Y) on the critical AMSTAR-2 items; total score= total score of 0 (N), 0.5 (PY), and 1 (Y)

**All AMSTAR-2 items.**

**Item 1:** Did the research questions and inclusion criteria for the review include the components of PI(E)CO (Population, Intervention or Exposure, Comparator, Outcome)?

**Item 2*:** Did the report of the review contain an explicit statement that the review methods were established prior to conduct of the review and did the report justify any significant deviations from the protocol?

**Item 3:** Did the review authors explain their selection of the study designs for inclusion in the review?

**Item 4*:** Did the review authors use a comprehensive literature search strategy?

**Item 5:** Did the review authors perform study selection in duplicate?

**Item 6:** Did the review authors perform data extraction in duplicate?

**Item 7*:** Did the review authors provide a list of excluded studies and justify the exclusions?

**Item 8:** Did the review authors describe the included studies in adequate detail?

**Item 9*:** Did the review authors use a satisfactory technique for assessing the risk of bias (RoB) in individual studies that were included in the review?

**Item 10:** Did the review authors report on the sources of funding for the studies included in the review?

**Item 11*:** If meta-analysis was justified did the review authors use appropriate methods for statistical combination of results?

**Item 12:** If meta-analysis was performed did the review authors assess the potential impact of RoB in individual studies on the results of the meta-analysis or other evidence synthesis?

**Item 13*:** Did the review authors account for RoB in individual studies when interpreting/ discussing the results of the review?

**Item 14:** Did the review authors provide a satisfactory explanation for, and discussion of, any heterogeneity observed in the results of the review?

**Item 15*:** If they performed quantitative synthesis did the review authors carry out an adequate investigation of publication bias (small study bias) and discuss its likely impact on the results of the review?

**Item 16:** Did the review authors report any potential sources of conflict of interest, including any funding they received for conducting the review?

**Table 9** **Details of evidence grading for significant associations from meta-analyses.**

| Factors | cases | P-value | 95%PI | I^2^ (%) | Egger’s P value | P | 95%PI | Heterogeneity | Cases | Egger’s P value | Evidence Convincing=1; highly suggestive=2; suggestive=3; weak=4 | Reason:  H=heterogeneity; B=bias; N=number of cases; P=P value; L=large |
| --- | --- | --- | --- | --- | --- | --- | --- | --- | --- | --- | --- | --- |
| Genetic factors（N=3） | | | | | | | | | | | | |
| allele D | 1348 | <0.05 | 1.37-2.12 | 0 | NA | <0.05 | excluding 1 | <50% | >1000 | NA | 3 | P |
| co-dominant model | 438 | <0.05 | 1.40-3.10 | 0 | NA | <0.05 | excluding 1 | <50% | <1000 | NA | 4 | N |
| recessive model | 674 | <0.05 | 1.83-3.54 | 0 | NA | <0.05 | excluding 1 | <50% | <1000 | NA | 4 | N |
| Viral infection（N=5） | | | | | | | | | | | | |
| All viruses | 1287 | 0.001 | 1.61-7.52 | 76 | 0.001 | <0.001 | excluding 1 | >50% | >1000 | <0.05 | 3 | H、B |
| EBV | 252 | <0.001 | 4.22-22.91 | 41.5 | 0.03 | <0.001 | excluding 1 | <50% | <1000 | <0.05 | 4 | N |
| CMV | 209 | 0.006 | 1.29-4.66 | 24.1 | 0.355 | <0.05 | excluding 1 | <50% | <1000 | >0.1 | 4 | N |
| HHV7 | 95 | 0.022 | 1.14-5.40 | 0 | 0.702 | <0.05 | excluding 1 | <50% | <1000 | >0.1 | 4 | N |
| HHV8 | 152 | <0.001 | 2.70-29.14 | 0 | 0.508 | <0.001 | excluding 1 | <50% | <1000 | >0.05 | 4 | N |
| Occupational and environmental exposures（N=6） | | | | | | | | | | | | |
| general dust | 2208 | <0.05 | 1.08-1.63 | 0 | >0.05 | <0.05 | excluding 1 | <50% | >1000 | >0.05 | 3 | P |
| wood | 264189 | <0.05 | 1.02-1.71 | 26.34 | >0.05 | <0.05 | excluding 1 | <50% | >1000 | >0.05 | 3 | P |
| metals | 13277 | <0.05 | 1.05-1.92 | 66.4 | 0.53 | <0.05 | excluding 1 | >50% | >1000 | >0.1 | 3 | P&H&N |
| pesticide exposure | 1446 | 0.005 | 1.24-3.45 | 0 | NA | <0.05 | excluding 1 | <50% | >1000 | NA | 3 | P |
| farming or agriculture workers | 1631 | 0.009 | 1.17-3.04 | 67 | NA | <0.05 | excluding 1 | >50% | >1000 | NA | 3 | P&H&N |
| increase in O3 exposure concentration | 628 | 0.001 | 1.16-1.83 | 0 | NA | <0.001 | excluding 1 | <50% | <1000 | NA | 4 | N |
| Lifestyle（N=2） | | | | | | | | | | | | |
| smoking | 8514 | <0.001 | 1.27-1.30 | 82.2 | >0.05 | <0.001 | excluding 1 | >50% | >1000 | >0.05 | 3 | H |
| former smokers | 6907 | <0.001 | 1.46-2.81 | 84.5 | >0.05 | <0.001 | excluding 1 | >50% | >1000 | >0.05 | 3 | H |
| Clinical variables（N=6） | | | | | | | | | | | | |
| GERD | 12574 | <0.0001 | 1.95-4.42 | 86 | NA | <0.001 | excluding 1 | >50% | >1000 | NA | 3 | H |
| Diabetes mellitus | 24191 | <0.0001 | 1.30-2.10 | 68 | 0.016 | <0.001 | excluding 1 | >50% | >1000 | <0.05 | 3 | H & B |
| FVC decreases by 10% or more | 270 | <0.001 | 1.82-7.03 | 0 | NA | <0.001 | excluding 1 | <50% | <1000 | NA | 4 | N |
| mMRC ≥ 2 | 760 | <0.001 | 1.38-1.90 | 30.6 | 0.202 | <0.05 | excluding 1 | <50% | <1000 | NA | 4 | N |
| %FVC increases by 10% | 536 | 0.001 | 0.74-0.92 | 19.3 | NA | <0.001 | excluding 1 | <50% | <1000 | NA | 4 | N |
| %FVC | 2473 | 0.001 | 0.96-0.99 | 65.7 | 0.118 | <0.001 | excluding 1 | >50% | >1000 | >0.05 | 3 | P&H&N |

Abbreviations: GERD: Gastroesophageal reflux disease; EBV: Epstein-Barr virus; CMV: CytoMegalo virus; HHV7: Human herpesvirus 7; HHV8: Human herpesvirus 8; FVC: Forced vital capacity; mMRC: modified medical research council; PI: prediction interval.

**Table 10** **Basic characteristics of included MR studies and evidence grading results.**

| Author, year | Exposure | Exposure sample size | Number of IVs | F statistic | Outcome cases/controls | Summary of metric  by IVW | Main analysis p-value | Quality  (Sensitivity analysis) | Evidence grading |
| --- | --- | --- | --- | --- | --- | --- | --- | --- | --- |
| Lifestyle (N=5) | | | | | | | | | |
| Wu et al. 2023 | Age of smoking initiation | 341427  Liu et al. 2019 | 16 | NA | 1812/338784  FinnGen | 0.108 (0.029, 0.402) | 0.001 | Yes | Suggestive |
|  | Maternal smoking | 397732  Ben Elsworth 2018 | 40 | NA | 1812/338784  FinnGen | 13.183 (1.82, 95.184) | 0.011 | Yes | Suggestive |
| Zhu et al. 2024 | Smoking initiation | NA | 378 | NA | 10382/ 968080  the International IPF Genetics Consortium +Global Biobank Meta-analysis Initiative | 1.280 (1.100, 1.490) | 0.002 | Yes | Probable |
|  | Lifetime smoking | NA | 126 | NA | 10382/ 968080  the International IPF Genetics Consortium +Global Biobank Meta-analysis Initiative | 1.620 (1.250, 2.090) | <0.001 | Yes | Probable |
| Lei et al. 2023 | Moderate-to-vigorous physical activity | GWAS Catalog | 18 | NA | 2018/373064  FinnGen Biobank | 0.270 (0.090, 0.820) | 0.020 | Yes | Probable |
| Diet and nutrition (N=6) | | | | | | | | | |
| Lai et al. 2023 | Pork intake | 460162  UK Biobank | 13 | NA | 198014  FinnGen Biobank | 1.05E+02  (4.354, 2.536E+03) | 0.004 | Yes | Probable |
| Zhang et al. 2024 | Oily fish intake | 460443  ukb-b-2209 | 49 | 38.93 | 1369  ebi-a-GCST90018120 | 0.996 (0.993, 0.998) | 0.001 | Yes | Suggestive |
|  | Yogurt intake | 64949  ukb-b-7753 | 13 | 21 | 1369  ebi-a-GCST90018120 | 0.997 (0.994, 1.000) | 0.020 | Yes | Robust |
|  | Average weekly beer  plus cider intake | 327634  ukb-b-5174 | 15 | 19.64 | 1369  ebi-a-GCST90018120 | 1.008 (1.001, 1.013) | 0.008 | Yes | Suggestive |
|  | Beef intake | 461053  ukb-b-2862 | 11 | 26.11 | 1369  ebi-a-GCST90018120 | 1.008 (1.001, 1.016) | 0.037 | Yes | Robust |
|  | Alcoholic drinks perweek | 335394  ieu-b-73 | 26 | 70.99 | 1369  ebi-a-GCST90018120 | 1.004 (1.001, 1.006) | 0.018 | Yes | Suggestive |
| Anthropometric indices (N=6) | | | | | | | | | |
| Ma et al. 2022 | BMI | 681275  Genetic Investigation of ANthropometric  Traits(GIANT) | 469 | 81.87 | 2668/8519  Meta-analysis | 1.510 (1.220, 1.870) | 1.27E-04 | Yes | Probable |
|  | WC | 232101  Genetic Investigation of ANthropometric  Traits (GIANT) | 40 | 37.12 | 2668/8519  Meta-analysis | 1.710 (1.080, 2.720) | 2.33E-02 | Yes | Probable |
| Cotton et al. 2023 | BMI | 694949  GWAS | 543 | 72 | 2668/8519  Meta-analysis | 1.520 (1.280, 1.820) | <0.010 | Yes | Insufficient |
| Jiang et al. 2023 | Weight | ukb-a-249 | 291 | NA | Allen.et.al | 1.440 (1.160, 1.780) | 8.71E-04 | Yes | Probable |
|  | BMI | ieu-b-40 | 442 | NA | Allen.et.al | 1.350 (1.130, 1.620) | 1.00E-03 | Yes | Probable |
|  | Whole body fat mass | ukb-a-265 | 248 | NA | Allen.et.al | 1.400 (1.140, 1.740) | 1.72E-03 | Yes | Probable |
|  | WC | ukb-a-382 | 195 | NA | Allen.et.al | 1.540 (1.160, 2.050) | 3.08E-03 | Yes | Probable |
|  | Trunk fat mass | ukb-a-291 | 248 | NA | Allen.et.al | 1.350 (1.100, 1.650) | 3.45E-03 | Yes | Probable |
|  | Body fat percentage | ukb-a-264 | 219 | NA | Allen.et.al | 1.550 (1.150, 2.080) | 3.86E-03 | Yes | Probable |
| Wu et al. 2023 | BMI | 681275  Yengo et al. 2018 | 403 | NA | 1812/338784  FinnGen | 1.425 (1.114, 1.823) | 0.005 | Yes | Suggestive |
|  | Body fat percentage | 454633  Ben Elsworth 2018 | 294 | NA | 1812/338784  FinnGen | 1.702 (1.202, 2.409) | 0.003 | Yes | Robust |
| Biomarkers (N=86) | | | | | | | | | |
| Duckworth et al. 2021 | Telomere length | NA | NA | NA | 1133/366942  UK Biobank | 4.190 (2.330, 7.550) | 0.031 | Yes | Insufficient |
| Nakanishi et al. 2022 | FUT3 | 3301  Proteome GWAS | NA | NA | 2668/8591  Meta-analysis | 0.810 (0.740, 0.880) | 6.30E-07 | Yes | Robust |
|  | FUT5 | 3301  Proteome GWAS | NA | NA | 2668/8591  Meta-analysis | 0.760 (0.680, 0.860) | 1.10E-05 | Yes | Robust |
| Jiang et al. 2023 | Apoliprotein B | ukb-d-30640 | 115 | NA | Allen.et.al | 0.780 (0.650, 0.930) | 5.47E-03 | Yes | Probable |
| Huang et al. 2023 | Circulating adiponectin levels | 39883  meta-analysis | 13 | 9.35 | 1028/196986  FinnGen Biobank | 0.645 (0.457, 0.911) | 0.013 | Yes | Robust |
| Jia et al. 2023 | IL-14 | NA | NA | NA | 1369/435866  Meta-analysis | 1.001 (1.000, 1.001) | 0.026 | Yes | Probable |
| Leavy et al. 2023 | Seropositive RA | 18019  Northwestern European:  、Sweden、Denmark、  Iceland、Norway、UK Biobank、FinnGen | 70 | NA | 4125/20464  European：UK Study、  Chicago Study、Colorado Study、UUS Study、Genentech | 0.930 (0.870, 0.990) | 0.032 | Yes | Robust |
| Liu et al. 2023 | Telomere length | 472174  UK Biobank | NA | NA | 1514/ 306603  FinnGen | 0.410 (0.290, 0.580) | 4.11E-07 | Yes | Robust |
| Shi et al. 2023 | Gut Microbiota-Family Coriobacteriia | NA | 13 | 20.94 | 6257/ 947616  GBMI | 0.763 (0.602, 0.968) | 0.026 | Yes | Probable |
|  | Gut Microbiota-Family Acidaminococcaceae | NA | 5 | 21.27 | 6257/ 947616  GBMI | 0.735 (0.552, 0.979) | 0.035 | Yes | Probable |
|  | Gut Microbiota-Family Coriobacteriaceae | NA | 13 | 20.94 | 6257/ 947616  GBMI | 0.763 (0.602, 0.998) | 0.026 | Yes | Probable |
|  | Gut Microbiota-Family XIII | NA | 10 | 21.18 | 6257/ 947616  GBMI | 0.782 (0.613, 0.998) | 0.048 | Yes | Probable |
|  | Gut Microbiota-Genus Ruminococcus gnavus | NA | 7 | 22.49 | 6257/ 947616  GBMI | 0.792 (0.629, 0.996) | 0.046 | Yes | Probable |
|  | Gut Microbiota-  Genus Blautia | NA | 10 | 21.5 | 6257/ 947616  GBMI | 1.269 (1.029, 1.565) | 0.026 | Yes | Probable |
|  | Gut Microbiota-  Genus Eisenbergiella | NA | 10 | 21.12 | 6257/ 947616  GBMI | 1.232 (1.075, 1.412) | 0.003 | Yes | Suggestive |
|  | Gut Microbiota-  Genus Holdemania | NA | 14 | 22.05 | 6257/ 947616  GBMI | 1.271 (1.095, 1.476) | 0.002 | Yes | Probable |
|  | Gut Microbiota-  Genus Hungatella | NA | 3 | 19.95 | 6257/ 947616  GBMI | 0.778 (0.629, 0.962) | 0.021 | Yes | Suggestive |
|  | Gut Microbiota-  Order Coriobacteriales | NA | 13 | 20.94 | 6257/ 947616  GBMI | 0.763 (0.602, 0.968) | 0.026 | Yes | Probable |
| Wu et al. 2023 | Telomere length | 472174  Codd et al. 2021 | 85 | NA | 1812/338784  FinnGen | 0.475 (0.336, 0.670) | <0.001 | Yes | Robust |
| Zhang et al. 2023 | Circulating CRP levels | 204402  a large-scale GWAS meta-analysis of 88 studies | 54 | NA | 1028/196896  FinnGen biobank | 1.446 (1.128, 1.854) | 0.004 | Yes | Probable |
| Fan et al. 2024 | Gut Microbiota-  Phylum Cyanobacteria | 18340  MiBioGen | 8 | NA | 1028/196896  FinnGen biobank | 1.631 (1.035, 2.571) | 0.035 | Yes | Probable |
|  | Gut Microbiota-  Family Bacteroidaceae | 18340  MiBioGen | 9 | NA | 1028/196896  FinnGen biobank | 1.917 (1.083, 3.393) | 0.026 | Yes | Probable |
|  | Gut Microbiota-  Family XIII | 18340  MiBioGen | 9 | NA | 1028/196896  FinnGen biobank | 0.452 (0.249, 0.820) | 0.009 | Yes | Probable |
|  | Gut Microbiota-  Order Selenomonadales | 18340  MiBioGen | 12 | NA | 1028/196896  FinnGen biobank | 0.563 (0.337, 0.941) | 0.029 | Yes | Probable |
|  | Gut Microbiota-  Order Gastranaerophilales | 18340  MiBioGen | 9 | NA | 1028/196896  FinnGen biobank | 1.441 (1.019, 2.036) | 0.039 | Yes | Probable |
|  | Gut Microbiota-  Genus Senegalimassilia | 18340  MiBioGen | 5 | NA | 1028/196896  FinnGen biobank | 2.280 (1.174, 4.427) | 0.015 | Yes | Probable |
|  | Gut Microbiota-  Genus Veillonella | 18340  MiBioGen | 6 | NA | 1028/196896  FinnGen biobank | 0.546 (0.304, 0.982) | 0.043 | Yes | Suggestive |
|  | Gut Microbiota-  Genus Ruminococcus gnavus | 18340  MiBioGen | 12 | NA | 1028/196896  FinnGen biobank | 0.717 (0.527, 0.976) | 0.034 | Yes | Probable |
|  | Gut Microbiota-  Genus.Oscillibacter | 18340  MiBioGen | 13 | NA | 1028/196896  FinnGen biobank | 0.571 (0.405, 0.806) | 0.001 | Yes | Probable |
| Ren et al. 2024 | Gut Microbiota-  Order Bifidobacteriales | 18340  MiBioGen | 16 | NA | 4125/20464  The Collaborative Group of genetic studies of IPF | 0.773 (0.610, 0.979) | 0.033 | Yes | Probable |
|  | Gut Microbiota-  Family Bifidobacteriaceae | 18340  MiBioGen | 16 | NA | 4125/20464  The Collaborative Group of genetic studies of IPF | 0.773 (0.610, 0.979) | 0.033 | Yes | Probable |
|  | Gut Microbiota-  Family Ruminococcaceae UCG009 | 18340  MiBioGen | 13 | NA | 4125/20464  The Collaborative Group of genetic studies of IPF | 0.793 (0.652, 0.965) | 0.020 | Yes | Probable |
|  | Gut Microbiota-  Genus Coprococcus2 | 18340  MiBioGen | 12 | NA | 4125/20464  The Collaborative Group of genetic studies of IPF | 1.349 (1.021, 1.783) | 0.035 | Yes | Probable |
| Wu et al. 2024 | Adiponectin | 32161  IEU OpenGWAS project:ieu-a-1 | 15 | NA | 198014  GWAS ID: fInn-b-IPF | 0.720 (0.520, 0.990) | 0.049 | Yes | Probable |
| Xu et al. 2024 | Testosterone | 353805  IEU GWAS database:ebi-a-GCST90014013 | 83 | NA | 198014  finn-b-IPF | 0.413 (0.187, 0.912) | 0.029 | Yes | Probable |
|  | Serum estrogen sulfotransferase | 3301  IEU GWAS database:prot-a-2892 | 3 | NA | 198014  finn-b-IPF | 1.320 (1.080,1.620) | 0.010 | Yes | Probable |
| Xu et al. 2024 | Glutamine levels | 114751  GWAS | 32 | NA | 2189/407609  FinnGen | 0.750 (0.592,0.951) | 0.017 | Yes | Robust |
| Zou et al. 2024 | Lipid-Carnitine | 7797  Shin et al. | 224 | NA | 435866  Meta-analysis | (0.992, 0.998) | 0.003 | Yes | Probable |
|  | Lipid-1-myristoylglycerophosphocholine | 7812  Shin et al. | 25 | NA | 435866  Meta-analysis | (1.002, 1.008) | 0.044 | Yes | Probable |
|  | Lipid-1-linoleoylglycerophosphoethanolamine | 7817  Shin et al. | 13 | NA | 435866  Meta-analysis | (0.993, 0.999) | 0.027 | Yes | Probable |
|  | Lipid-1-eicosatrienoylglycerophosphocholine | 7809  Shin et al. | 20 | NA | 435866  Meta-analysis | (0.994, 0.998) | 0.017 | Yes | Probable |
|  | Lipid-Total cholesterol in large HDL | 21558  Shin et al. | 68 | NA | 435866  Meta-analysis | (1.000, 1.001) | 0.006 | Yes | Probable |
|  | Lipid-Cholesterol esters in very large HDL | 19273  Shin et al. | 45 | NA | 435866  Meta-analysis | (1.000, 1.001) | 0.023 | Yes | Probable |
|  | Lipid-Phospholipids in very large HDL | 19273  Shin et al. | 53 | NA | 435866  Meta-analysis | (1.000, 1.001) | 0.005 | Yes | Probable |
|  | Nucleotide-Hypoxanthine | 7287  Shin et al. | 25 | NA | 435866  Meta-analysis | (0.993, 0.999) | 0.003 | Yes | Probable |
|  | Ratio of omega-6 fatty acids to omega-3 fatty acids | 114999  Shin et al. | 167 | NA | 435866  Meta-analysis | (0.999, 1.000) | 0.004 | Yes | Probable |
|  | Degree of unsaturation | 114999  Shin et al. | 202 | NA | 435866  Meta-analysis | (1.001, 1.001) | 0.021 | Yes | Probable |
|  | Phospholipids to total lipids ratio in chylomicrons and extremely | 111631  Shin et al. | 152 | NA | 435866  Meta-analysis | (0.998, 0.999) | 0.017 | Yes | Probable |
|  | Large VLDL | 7178  Shin et al. | 44 | NA | 435866  Meta-analysis | (1.001, 1.008) | 0.039 | Yes | Probable |
| Wu et al. 2024 | Gut Microbiota-  Genus Actinomycetes | 492803  MiBioGen | 22 | 31.584 | 198014  Finn-b-IPF | 1.773 (1.323, 2.377) | <0.001 | Yes | Suggestive |
|  | Gut Microbiota-  Genus Erysipelatoclostridium | 492803  MiBioGen | 3 | 33.461 | 198014  Finn-b-IPF | 2.077 (1.107, 3.896) | 0.023 | Yes | Suggestive |
|  | Gut Microbiota-  Genus Streptococcus | 492803  MiBioGen | 63 | 32.745 | 198014  Finn-b-IPF | 1.350 (1.100, 1.657) | 0.004 | Yes | Suggestive |
|  | Gut Microbiota-  Genus Bifidobacterium | 492803  MiBioGen | 249 | 33.781 | 198014  Finn-b-IPF | 0.668 (0.620, 0.720) | <0.001 | Yes | Probable |
|  | Gut Microbiota-  Genus Ruminococcus | 492803  MiBioGen | 5 | 30.691 | 198014  Finn-b-IPF | 0.434 (0.222, 0.848) | 0.015 | Yes | Suggestive |
|  | Gut Microbiota-  Genus Tyzzerella | 492803  MiBioGen | 3 | 33.329 | 198014  Finn-b-IPF | 0.479 (0.304, 0.755) | 0.001 | Yes | Suggestive |
| Wu et al. 2024 | Gut Microbiota-  Class Bacilli | 492803  MiBioGen | 6 | NA | 392423  Finngen- R9-IPF | 1.340 (1.030, 1.760) | 0.03 | Yes | Probable |
|  | Gut Microbiota-  Class Negativicutes | 492803  MiBioGen | 12 | NA | 392423  Finngen- R9-IPF | 1.430 (1.000, 2.050) | 0.049 | Yes | Probable |
|  | Gut Microbiota-Order Burkholderiales | 492803  MiBioGen | 8 | NA | 392423  Finngen- R9-IPF | 1.650 (1.230, 2.220) | 0.001 | Yes | Probable |
|  | Gut Microbiota-  Family XIII | 492803  MiBioGen | 7 | NA | 392423  Finngen- R9-IPF | 1.660 (1.030, 2.680) | 0.039 | Yes | Probable |
|  | Gut Microbiota-  Genus Ruminococcus gnavus | 492803  MiBioGen | 11 | NA | 392423  Finngen- R9-IPF | 1.360 (1.080, 1.700) | 0.008 | Yes | Probable |
|  | Gut Microbiota-  Genus Methanobrevibacter | 492803  MiBioGen | 11 | NA | 392423  Finngen- R9-IPF | 1.410 (1.010, 1.960) | 0.044 | Yes | Probable |
|  | Gut Microbiota-  Species Alistipes finegoldii | 492803  MiBioGen | 3 | NA | 392423  Finngen- R9-IPF | 1.400 (1.080, 1.800) | 0.010 | Yes | Probable |
|  | Gut Microbiota-  Genus Eisenbergiella | 492803  MiBioGen | 9 | NA | 392423  Finngen- R9-IPF | 0.800 (0.640, 0.990) | 0.037 | Yes | Suggestive |
| Li et al. 2024 | CD39 on CD39+  CD8+ T cells | 3757  IEU Open GWAS | 32 | NA | 2018/373064  Finnish Genetic Consortium | 0.850 (0.787, 0.918) | 3.68E-05 | Yes | Probable |
|  | CD4 on HLA DR+ CD4+ T cell | 3757  IEU Open GWAS | 26 | NA | 2018/373064  Finnish Genetic Consortium | 0.897 (0.821, 0.981) | 0.018 | Yes | Probable |
|  | CD4 on activated CD4 regulatory T cell | 3757  IEU Open GWAS | 29 | NA | 2018/373064  Finnish Genetic Consortium | 0.950 (0.909, 0.994) | 0.025 | Yes | Probable |
|  | CD8 on Terminally Differentiated CD8+Tcell | 3757  IEU Open GWAS | 20 | NA | 2018/373064  Finnish Genetic Consortium | 1.124 (1.029, 1.226) | 0.009 | Yes | Robust |
|  | CD25 on activated CD4 regulatory T cell | 3757  IEU Open GWAS | 19 | NA | 2018/373064  Finnish Genetic Consortium | 0.868 (0.769, 0.981) | 0.023 | Yes | Probable |
|  | BAFF-R on transitional B cell | 3757  IEU Open GWAS | 96 | NA | 2018/373064  Finnish Genetic Consortium | 0.956 (0.924, 0.990) | 0.011 | Yes | Probable |
|  | CD24 on IgD- CD38dim B cell | 3757  IEU Open GWAS | 7 | NA | 2018/373064  Finnish Genetic Consortium | 0.825 (0.699, 0.973) | 0.022 | Yes | Probable |
|  | FSC-A on Natural Killer | 3757  IEU Open GWAS | 22 | NA | 2018/373064  Finnish Genetic Consortium | 1.102 (1.021, 1.190) | 0.013 | Yes | Probable |
| Zhang et al. 2024 | Herpes simplex virus infection | 1595+211856  finn-b | 9 | NA | 1028/196986  finn-b-IPF | 1.280 (1.048, 1.563) | 0.015 | Yes | Probable |
| He et al. 2024 | CD33br HLA DR+ CD14dim AC | 3757  Immunity-wide GWAS data sources GWAS Catalog | 27 | NA | 6257/947616  meta-analysis | 0.960 (0.930, 0.990) | 0.007 | Yes | Probable |
|  | CD14+ CD16+ monocyte %monocyte | 3757  Immunity-wide GWAS data sources GWAS Catalog | 20 | NA | 6257/947616  meta-analysis | 0.935 (0.880, 0.990) | 0.024 | Yes | Suggestive |
|  | CD14+ CD16- monocyte %monocyte | 3757  Immunity-wide GWAS data sources GWAS Catalog | 20 | NA | 6257/947616  meta-analysis | 1.048 (1.010, 1.090) | 0.011 | Yes | Probable |
|  | CD4/CD8br | 3757  Immunity-wide GWAS data sources GWAS Catalog | 14 | NA | 6257/947616  meta-analysis | 1.120 (1.030, 1.220) | 0.010 | Yes | Probable |
|  | CD8dim %T cell | 3757  Immunity-wide GWAS data sources GWAS Catalog | 14 | NA | 6257/947616  meta-analysis | 1.120 (1.030, 1.220) | 0.010 | Yes | Probable |
|  | TCRgd %T cell | 3757  Immunity-wide GWAS data sources GWAS Catalog | 12 | NA | 6257/947616  meta-analysis | 0.931 (0.880, 0.980) | 0.007 | Yes | Probable |
|  | TCRgd %lymphocyte | 3757  Immunity-wide GWAS data sources GWAS Catalog | 15 | NA | 6257/947616  meta-analysis | 0.960 (0.920, 1.000) | 0.041 | Yes | Probable |
|  | CD8dim NKT %T cell | 3757  Immunity-wide GWAS data sources GWAS Catalog | 19 | NA | 6257/947616  meta-analysis | 1.111 (1.040, 1.180) | 0.001 | Yes | Probable |
|  | CD8dim NKT %lymphocyte | 3757  Immunity-wide GWAS data sources GWAS Catalog | 26 | NA | 6257/947616  meta-analysis | 1.080 (1.020, 1.140) | 0.005 | Yes | Probable |
|  | DN (CD4-CD8-) NKT %T cell | 3757  Immunity-wide GWAS data sources GWAS Catalog | 30 | NA | 6257/947616  meta-analysis | 1.079 (1.020, 1.140) | 0.007 | Yes | Suggestive |
|  | HLA DR+ NK AC | 3757  Immunity-wide GWAS data sources GWAS Catalog | 16 | NA | 6257/947616  meta-analysis | 0.907 (0.850, 0.970) | 0.003 | Yes | Probable |
|  | CD39+ CD8br %T cell | 3757  Immunity-wide GWAS data sources GWAS Catalog | 18 | NA | 6257/947616  meta-analysis | 0.929 (0.870, 0.990) | 0.018 | Yes | Probable |
|  | CD19 on IgD+ CD38- naïve | 3757  Immunity-wide GWAS data sources GWAS Catalog | 15 | NA | 6257/947616  meta-analysis | 0.954 (0.910, 1.000) | 0.038 | Yes | Probable |
|  | CD3 on activated & secreting Treg | 3757  Immunity-wide GWAS data sources GWAS Catalog | 20 | NA | 6257/947616  meta-analysis | 0.937 (0.890, 0.980) | 0.009 | Yes | Probable |
|  | CD3 on CD28- CD8br | 3757  Immunity-wide GWAS data sources GWAS Catalog | 13 | NA | 6257/947616  meta-analysis | 0.912 (0.850, 0.980) | 0.015 | Yes | Probable |
|  | PDL-1 on CD14- CD16+ monocyte | 3757  Immunity-wide GWAS data sources GWAS Catalog | 16 | NA | 6257/947616  meta-analysis | 0.916 (0.860, 0.970) | 0.003 | Yes | Probable |
|  | CD45 on CD33br HLA DR+ CD14- | 3757  Immunity-wide GWAS data sources GWAS Catalog | 17 | NA | 6257/947616  meta-analysis | 1.066 (1.010, 1.120) | 0.019 | Yes | Probable |
|  | CD45 on Mo MDSC | 3757  Immunity-wide GWAS data sources GWAS Catalog | 15 | NA | 6257/947616  meta-analysis | 1.072 (1.020, 1.130) | 0.009 | Yes | Probable |
|  | SSC-A on lymphocyte | 3757  Immunity-wide GWAS data sources GWAS Catalog | 18 | NA | 6257/947616  meta-analysis | 0.899 (0.830, 0.980) | 0.013 | Yes | Probable |
|  | CD45RA on naive CD8br | 3757  Immunity-wide GWAS data sources GWAS Catalog | 17 | NA | 6257/947616  meta-analysis | 0.915 (0.860, 0.970) | 0.005 | Yes | Probable |
| Clinical variables (N=5) | | | | | | | | | |
| Cao et al. 2024 | Age at menarche | 29346  MRbase  database. | 9 | NA | 1369/435866  IPF GWAS | 1.004 (1.001, 1.002) | 0.001 | Yes | Probable |
| Zhang et al. 2021 | Hypothyroidism | 289307  UK Biobank | NA | NA | 2668/8591  IPF GWAS | 1.125 (1.028,1.231) | 0.011 | Yes | Robust |
| Reynolds et al. 2022 | GERD | 78707+288734  genetic data from the largest (UK and Australia) | 59 | NA | 4125/20464  Meta-analysis | 1.600 (1.040, 2.490) | 0.032 | Yes | Insufficient |
| Cheng et al. 2023 | GERD | 78707+288734  a meta-analysis of  GWASs in European populations | 58 | NA | 4125/20464  Meta-analysis | 1.430 (1.050, 1.950) | 0.022 | Yes | Probable |
| Cotton et al. 2023 | GERD | 80265+305011  meta-analysis | 24 | 34 | 2668/8591  Meta-analysis | 1.580 (1.100, 2.250) | 0.010 | Yes | Insufficient |
| Chen et al. 2024 | Psoriasis | 462933  (GWAS), including IEU openGWAS and NealELab. | 20 | NA | 1028/196986  FinnGen biobank | 1.090 (1.010, 1.180) | 0.020 | Yes | Probable |
| Kanget al. 2024 | Diabetes mellitus | 29652  ebi-a-GCST005536 | 35 | NA | 1028/196986  FinnGen Biobank | 1.118 (1.021, 1.225) | 0.016 | Yes | Probable |
| Ma et al .2024 | Diabetes mellitus | 18856  European Bioinformatics Institute | 37 | NA | 1028/196986  FinnGen Biobank | 1.113 (1.019, 1.217) | 0.017 | Yes | Probable |
| Wu et al. 2024 | GERD | 462753+1127474  GWAS Catalog（https://www.ebi.ac.uk/gwas/） | 76 | NA | 1369/ 435866 | 1.002 (1.001, 1.003) | <0.001 | Yes | Probable |
| Wang et al. 2024 | Psoriasis | 9267+364071  FinnGen release R9 | NA | NA | 2018/373064  FinnGen release R9 | 1.140 (1.080, 1.220) | <0.001 | Yes | Robust |

**Table 11** **Details of STROBE-MR guidelines for reporting quality of MR studies.**

| **Study** | **1** | **2** | **3** | **4** | **5** | **6** | **7** | **8** | **9** | **10** | **11** | **12** | **13** | **14** | **15** | **16** | **17** | **18** | **19** | **20** | **Total score** |
| --- | --- | --- | --- | --- | --- | --- | --- | --- | --- | --- | --- | --- | --- | --- | --- | --- | --- | --- | --- | --- | --- |
| Duckworth et al. 2021 | 1 | 1 | 1 | 1 | 1 | 1 | 1 | 1 | 1 | 1 | 1 | 1 | 1 | 1 | 1 | 1 | 0 | 1 | 1 | 1 | 19 (95%) |
| Zhang et al. 2021 | 1 | 1 | 1 | 1 | 1 | 1 | 1 | 1 | 1 | 1 | 1 | 1 | 1 | 1 | 1 | 1 | 0 | 1 | 1 | 1 | 19 (95%) |
| Ma et al. 2022 | 0 | 1 | 1 | 1 | 1 | 1 | 1 | 1 | 1 | 0 | 1 | 1 | 1 | 1 | 1 | 1 | 1 | 1 | 1 | 0 | 17 (85%) |
| Nakanishi et al. 2022 | 1 | 1 | 1 | 1 | 1 | 1 | 1 | 1 | 1 | 1 | 1 | 1 | 1 | 1 | 1 | 1 | 1 | 1 | 0 | 1 | 19 (95%) |
| Reynolds et al. 2022 | 1 | 1 | 1 | 1 | 1 | 1 | 1 | 1 | 1 | 1 | 1 | 1 | 1 | 1 | 1 | 1 | 1 | 1 | 0 | 1 | 19 (95%) |
| Cheng et al. 2023 | 1 | 1 | 1 | 1 | 0 | 1 | 1 | 1 | 1 | 1 | 1 | 1 | 1 | 1 | 1 | 1 | 1 | 0 | 1 | 1 | 18 (90%) |
| Huang et al. 2023 | 1 | 1 | 1 | 1 | 1 | 1 | 1 | 1 | 1 | 0 | 1 | 1 | 1 | 1 | 1 | 1 | 1 | 1 | 0 | 1 | 18 (90%) |
| Jia et al. 2023 | 1 | 1 | 1 | 1 | 1 | 1 | 1 | 1 | 1 | 0 | 1 | 1 | 1 | 1 | 1 | 1 | 1 | 0 | 0 | 1 | 17 (85%) |
| Jiang et al. 2023 | 1 | 1 | 1 | 1 | 1 | 1 | 1 | 1 | 1 | 1 | 1 | 1 | 1 | 1 | 1 | 1 | 1 | 1 | 1 | 1 | 20 (100%) |
| Lai et al. 2023 | 1 | 1 | 1 | 1 | 1 | 1 | 1 | 1 | 1 | 1 | 1 | 1 | 1 | 1 | 1 | 1 | 0 | 1 | 1 | 1 | 19 (95%) |
| Leavy et al. 2023 | 1 | 1 | 1 | 1 | 1 | 1 | 1 | 1 | 1 | 0 | 1 | 1 | 1 | 1 | 1 | 1 | 1 | 1 | 1 | 1 | 19 (95%) |
| Lei et al. 2023 | 1 | 1 | 1 | 1 | 1 | 1 | 1 | 1 | 1 | 0 | 1 | 1 | 1 | 1 | 1 | 1 | 1 | 1 | 1 | 1 | 19 (95%) |
| Liu et al. 2023 | 1 | 1 | 1 | 1 | 1 | 1 | 1 | 1 | 1 | 0 | 1 | 1 | 1 | 1 | 1 | 1 | 1 | 1 | 1 | 1 | 19 (95%) |
| Shi et al. 2023 | 1 | 1 | 1 | 1 | 1 | 1 | 1 | 1 | 1 | 1 | 1 | 1 | 1 | 1 | 1 | 1 | 1 | 1 | 1 | 1 | 20 (100%) |
| Wu et al. 2023 | 0 | 1 | 1 | 1 | 1 | 1 | 1 | 1 | 1 | 1 | 1 | 1 | 1 | 1 | 1 | 1 | 0 | 1 | 1 | 1 | 18 (90%) |
| Zhang et al. 2023 | 1 | 1 | 1 | 1 | 1 | 1 | 1 | 1 | 1 | 0 | 1 | 1 | 1 | 1 | 1 | 1 | 1 | 0 | 1 | 1 | 18 (90%) |
| Cao et al. 2024 | 1 | 1 | 1 | 1 | 1 | 1 | 1 | 1 | 1 | 0 | 1 | 1 | 1 | 1 | 1 | 1 | 1 | 0 | 0 | 1 | 17 (85%) |
| Chen et al. 2024 | 1 | 1 | 1 | 1 | 1 | 1 | 1 | 1 | 1 | 0 | 1 | 1 | 1 | 1 | 1 | 1 | 1 | 1 | 1 | 1 | 19 (95%) |
| Fan et al. 2024 | 1 | 1 | 1 | 1 | 1 | 1 | 1 | 1 | 1 | 0 | 1 | 1 | 1 | 1 | 1 | 1 | 1 | 0 | 1 | 1 | 18 (90%) |
| Kanget al. 2024 | 1 | 1 | 1 | 1 | 1 | 1 | 1 | 1 | 1 | 1 | 1 | 1 | 1 | 1 | 1 | 1 | 1 | 0 | 1 | 1 | 19 (95%) |
| Ma et al .2024 | 1 | 1 | 1 | 1 | 1 | 1 | 1 | 1 | 1 | 0 | 1 | 1 | 1 | 1 | 1 | 1 | 1 | 1 | 0 | 1 | 18 (90%) |
| Ren et al. 2024 | 1 | 1 | 1 | 1 | 1 | 1 | 1 | 1 | 1 | 1 | 1 | 1 | 1 | 1 | 1 | 1 | 1 | 1 | 1 | 1 | 20 (100%) |
| Wu et al. 2024 | 1 | 1 | 1 | 1 | 1 | 1 | 1 | 1 | 1 | 1 | 1 | 1 | 1 | 1 | 1 | 1 | 1 | 0 | 1 | 1 | 19 (95%) |
| Wu et al. 2024 | 0 | 1 | 1 | 1 | 1 | 1 | 1 | 1 | 1 | 0 | 1 | 1 | 1 | 1 | 1 | 1 | 1 | 0 | 1 | 1 | 17 (85%) |
| Xu et al. 2024 | 1 | 1 | 1 | 1 | 1 | 1 | 1 | 1 | 1 | 0 | 1 | 1 | 1 | 1 | 1 | 1 | 1 | 1 | 0 | 1 | 18 (90%) |
| Xu et al. 2024 | 1 | 1 | 1 | 1 | 1 | 1 | 1 | 1 | 0 | 0 | 1 | 1 | 1 | 1 | 1 | 1 | 0 | 1 | 1 | 1 | 17 (85%) |
| Zhang et al. 2024 | 1 | 1 | 1 | 1 | 1 | 1 | 1 | 1 | 1 | 1 | 1 | 1 | 1 | 1 | 1 | 1 | 1 | 1 | 0 | 1 | 19 (95%) |
| Zou et al. 2024 | 1 | 1 | 1 | 1 | 1 | 1 | 1 | 1 | 1 | 0 | 1 | 1 | 1 | 1 | 1 | 1 | 1 | 1 | 1 | 1 | 19 (95%) |
| Wu et al. 2024 | 1 | 1 | 1 | 1 | 1 | 1 | 1 | 1 | 1 | 0 | 1 | 1 | 1 | 1 | 1 | 1 | 1 | 1 | 0 | 1 | 18 (90%) |
| Wu et al. 2024 | 1 | 1 | 1 | 1 | 1 | 1 | 1 | 1 | 1 | 1 | 1 | 1 | 1 | 1 | 1 | 1 | 1 | 1 | 0 | 1 | 19 (95%) |
| Li et al. 2024 | 1 | 1 | 1 | 1 | 1 | 1 | 1 | 1 | 1 | 0 | 1 | 1 | 1 | 1 | 1 | 1 | 1 | 1 | 1 | 1 | 19 (95%) |
| Zhang et al. 2024 | 1 | 1 | 1 | 1 | 0 | 1 | 1 | 1 | 1 | 0 | 1 | 1 | 1 | 1 | 1 | 1 | 1 | 1 | 1 | 1 | 18 (90%) |
| Wang et al. 2024 | 1 | 1 | 1 | 1 | 1 | 1 | 1 | 1 | 1 | 1 | 1 | 1 | 1 | 1 | 1 | 1 | 1 | 1 | 0 | 1 | 19 (95%) |
| He et al. 2024 | 1 | 1 | 1 | 1 | 1 | 1 | 1 | 1 | 1 | 1 | 1 | 1 | 1 | 1 | 1 | 1 | 1 | 1 | 1 | 1 | 20 (100%) |
| Duckworth et al. 2021 | 1 | 1 | 1 | 1 | 1 | 1 | 1 | 1 | 1 | 1 | 1 | 1 | 1 | 1 | 1 | 1 | 0 | 1 | 1 | 1 | 19 (95%) |
| Zhang et al. 2021 | 1 | 1 | 1 | 1 | 1 | 1 | 1 | 1 | 1 | 1 | 1 | 1 | 1 | 1 | 1 | 1 | 0 | 1 | 1 | 1 | 19 (95%) |

**Title and Abstract**

**Item 1:** Title and Abstract

**Introduction**

**Item 2:** Background

**Item 3:** Objectives

**Methods**

**Item 4:** Study design and data sources

**Item 5:** Assumptions

**Item 6:** Statistical methods: main analysis

**Item 7:** Assessment of assumptions

**Item 8:** Sensitivity analyses and additional analyses

**Item 9:** Software and pre-registration

**Results**

**Item 10:** Descriptive data

**Item 11:** Main results

**Item 12:** Assessment of assumptions

**Item 13:** Sensitivity analyses and additional analyses

**Discussion**

**Item 14:** Key results

**Item 15:** Limitations

**Item 16:** Interpretation

**Item 17:** Generalizability

**Other information**

**Item 18:** Funding

**Item 19:** Data and data sharing

**Item 20:** Conflicts of Interest

**2 Supplementary Figures**

**Figure 1** Forest plot of studies that evaluated the causal effect between BMI and IPF.

**Figure 2** Forest plot of studies that evaluated the causal effect between WC and IPF**.**

**Figure 3** Forest plot of studies that evaluated the causal effect between body fat percentage and IPF.

**Figure 4** Forest plot of studies that evaluated the causal effect between telomere length and IPF.

**Figure 5** Forest plot of studies that evaluated the causal effect between gut microbiota-family XIII and IPF.

**Figure 6** Forest plot of studies that evaluated the causal effect between gut microbiota-genus ruminococcus gnavus and IPF.

**Figure 7** Forest plot of studies that evaluated the causal effect between GERD and IPF**.**

**Figure 8** Forest plot of studies that evaluated the causal effect between diabetes mellitus and IPF.

**Figure 9** Forest plot of studies that evaluated the causal effect between psoriasis and IPF.
